# RUCAM-ascertained immunology and autoimmunity specifics of six idiosyncratic drug-induced liver injury types with refined classification: their individual complex molecular interplay

**DOI:** 10.3389/fimmu.2026.1771858

**Published:** 2026-07-28

**Authors:** Rolf Teschke

**Affiliations:** Department of Internal Medicine II, Division of Gastroenterology and Hepatology, Klinikum Hanau, Academic Teaching Hospital of the Medical Faculty, Goethe University Frankfurt/Main, Hanau, Germany

**Keywords:** RUCAM, adaptive immune system, ALDEN, autoimmune hepatitis, DIAIH, idiosyncratic DILI, immune iDILI by ICIs

## Abstract

Idiosyncratic drug-induced liver injury (iDILI) is not a uniform disease but rather includes a variety of types based on immune, autoimmune, and clinical considerations. This review attempts to close information gaps regarding the role of immunity and autoimmunity involved in the different iDILI disease types. The analysis of the current literature focusing on iDILI reveals compelling evidence of a pivotal role of immunity or autoimmunity in various types of iDILI. Among the autoimmune-triggered ones are the drug-induced autoimmune hepatitis (DIAIH) and the idiosyncratic drug-induced anti-CYP autoimmune hepatitis. In contrast, clearly immune-triggered are the human leukocyte antigen (HLA)-based immune iDILI, the immune iDILI with Stevens–Johnson syndrome (SJS) and toxic epidermal necrolysis (TEN), and the immune iDILI induced by immune checkpoint inhibitors (ICIs), but immune-triggered mechanisms account for only a part of the classic iDILI cases. For all these iDILI types, the use of the original or updated Roussel Uclaf Causality Assessment Method (RUCAM) allowed for confirming causality of the implicated drug, assisted by the simplified autoimmune hepatitis (AIH) score in the DIAIH cases and the Algorithm of Drug Causality for Epidermal Necrolysis (ALDEN) score in the cases of immune-based iDILI with SJS/TEN. All these diagnostic causality assessment algorithms are validated methods and have helped define clinical features of the different iDILI types. The first treatment goal is the cessation of the suspected drug, which alone may lead to clinical and laboratory improvement and restoration of health. However, therapy with immunosuppressive agents is often needed to treat the injury caused by immune and autoimmune processes and can lead to complete remission in all iDILI types with the exception of the classic iDILI, where only a subset of the patients achieve remission. At the molecular pathomechanistic level, there is a complex interplay mostly related to the adaptive immune system activated by the innate system. In sum, immunology and autoimmunity specifics in cases of RUCAM-ascertained iDILI types, as part of a refined classification, remain a challenging topic that can be deepened by future cases if validated diagnostic algorithms are applied.

## 1 Introduction

Idiosyncratic drug-induced liver injury (iDILI) has recently received much attention because of immune and autoimmune variability involved in the emerging disease, suggesting that mechanistically and clinically, iDILI is not a uniform and homogeneous liver disease but rather consists of several types (1–4). This heterogeneity calls for a thorough evaluation of each type, primarily regarding individual causality assessment methods, to verify the diagnosis because some iDILI types consist of two parts that must be evaluated using different causality assessment methods in addition to the original Roussel Uclaf Causality Assessment Method (RUCAM) (5, 6) or its updated version (7), which are obligatory diagnostic methods applicable to all iDILI types (4, 8–10). In general, causality assessment methods facilitate the formation of homogeneous iDILI study cohorts by replacing difficult-to-evaluate heterogeneous study cohorts, which often contain cases due to alternative non-drug causes that are untreated to chemical medications implicated in iDILI types (10). Homogeneous iDILI-type cohorts help better characterize specific clinical features and pathogenetic steps, including not only simple immune reactions but also autoimmune mechanisms.

Of concern is the observation that clinical differentiation of iDILI types was often neglected, leading to incorrect final diagnoses (8). This neglect applies to case reports, case series, national European DILI case registries, the US DILI Network, and the US LiverTox database, in addition to numerous other worldwide databases focused on iDILI. Mixing all iDILI types is problematic in medical science and prevents accurate case and cohort characterization.

This analytical and critical review attempts to close existing information gaps related to iDILI types. The focus is on refined disease-type classification based on results obtained from causality assessments using validated diagnostic algorithms. An additional aim is to replace the heterogeneous iDILI types with homogeneous iDILI types.

## 2 Strategy of literature search

The literature search involved the PubMed database and Google Scholar. The following terms were used: DIAIH, autoimmune DILI, haptens, immune-mediated DILI, DILI with autoimmune features, DIALH, AIH anti-cytochrome P450 (CYP) antibodies, human leukocyte antigens (HLAs), Stevens–Johnson syndrome (SJS), and toxic epidermal necrosis (TEN). The search was completed on 22 November 2025. Preference was given to reports with cases evaluated using validated causality assessment methods such as RUCAM. Instead of using the validated RUCAM as the preferred diagnostic algorithm for causality assessment, different research networks and clinical groups have used various non-validated diagnostic frameworks; however, their shortcomings outweigh possible strengths. Considering these aspects and for reasons of balance, publications with cases evaluated using non-validated causality tools were also included in the compiled listings of various iDILI types, but due to a weak evidence base, their results remained undiscussed to avoid confounding issues. The search strategy focused on reports in the English language and was not restricted by time of publication. Appropriate cases were retrieved from published cases series, individual case reports, and review articles.

## 3 Classification and standardization

Conceptually, intrinsic drug-induced liver injury results from drugs used at doses above recommendations and must be differentiated from iDILI that develops after treatment with drugs used at recommended doses but lacks a clear dose dependence (7). Based on various validated causality assessment methods (5–7, 11, 12) and molecular pathomechanistic considerations, a conceptual framework of various iDILI types related to immune and autoimmune mechanisms underlying many iDILI cases was developed. Such iDILI typology will help clinicians, scientists, and regulators improve the clinical management of iDILI patients and epidemiological aspects. These conceptual considerations led to a range of compiled listings of iDILI types related to immune or autoimmune processes, as provided in the brief overview (**Table 1**).

**Table 1:** Listing of iDILI types.

Definition	Immunity or autoimmunity	Characteristic case features and treatment efficacy	Causality assessment
Classic idiosyncratic DILI (iDILI)	Immunity ±	Lack of serum autoimmune parameters but in a few iDILI cases, liver histology signified immunology. Response to immunosuppressants was ineffective in some iDILI patients, possibly reflecting lacking immune involvement in these cases.	RUCAM (5–7)
Drug-induced autoimmune hepatitis (DIAIH)	Autoimmunity +	Combined features of iDILI plus those of AIH with increased titers of serum autoimmune parameters. Complete remission with immunosuppressants achievable.	RUCAM (5–7) combined with the simplified AIH score (11)
Idiosyncratic drug-induced anti-CYP autoimmune hepatitis	Autoimmunity +	Autoimmunity verified by detection of serum anti-CYP antibodies. Complete remission following therapy with immunosuppressant agents is achievable.	RUCAM (5–7)
HLA-based immune iDILI	Immunity +	Serum human leukocyte antigens (HLA) and signs of immunity in serum and liver. Complete remission under immunosuppressants is achievable.	RUCAM (5–7)
Immune iDILI with SJS/TEN	Immunity +	A continuous iDILI disease spectrum with the Stevens- Johnson syndrome (SJS), the milder form as compared with the toxic epidermal necrolysis (TEN), the more serious one. Complete remission using immunosuppressant agents is achievable.	RUCAM (5–7) for DILI and the ALDEN score (12) for SJS and TEN
Immune iDILI by ICIs	Immunity +	Immune checkpoint inhibitors represent monoclonal antibodies and are used to treat patients with cancer. Apart from clinical efficacy, immune based iDILI can develop. Complete remission under treatment with immunosuppressant agents is achievable.	RUCAM (5–7)

Currently, six iDILI types have been identified, all with verified causality by RUCAM alone or in combination with another diagnostic algorithm; all algorithms used were validated methods (11, 12, 57). This list of immune iDILI types was modified and derived from a previous report published in an open-access journal (4). Treatment efficacy refers to outcomes after drug cessation. Abbreviations: ALDEN, Algorithm of Drug Causality for Epidermal Necrolysis; AIH, Autoimmune Hepatitis; CYP, cytochrome P450; ICI, immune checkpoint inhibitor; iDILI, idiosyncratic drug-induced liver injury; RUCAM, Roussel Uclaf Causality Assessment Method; SJS, Stevens–Johnson syndrome; TEN, toxic epidermal necrolysis.

## 4 Causality assessment algorithm methods used for iDILI types

### 4.1 RUCAM

RUCAM in its original version (5, 6) or, preferably, its 2016 update (7), are the preferred standard tools evaluating causality for suspected drugs (9, 10). The advantages of RUCAM include internal method validation (6), supported by subsequent external validation (13–15), as summarized (9). RUCAM is recognized by the US LiverTox database, which classifies the RUCAM system as a method of assigning points for clinical, serological, biochemical, and radiological characteristics of liver injury (16). The database also details that the RUCAM system provides an overall assessment score reflecting the likelihood that hepatic injury is attributable to a specific medication (16). In addition, it confirmed that RUCAM is now widely used to assess causality in DILI, both in the published literature and in support of regulatory decisions regarding medications implicated in hepatic injury. The LiverTox database specifies that RUCAM has been evaluated for accuracy, reproducibility, and intraobserver variability. Because the RUCAM score is based on objective criteria, there should be little or no variation in final scores obtained by different investigators, as clarified by the LiverTox database (16). Indeed, RUCAM is also known for its transparency, liver injury specificity, objectivity (5, 7, 9), worldwide use with top ranking (17), and its scoring system, which provides causality gradings from excluded to highly probable (7, 9). Limitations have been described when assessors neglect the principles of good clinical practice and manipulate RUCAM scores (9). Such unscientific management attempts, as uncovered and pleaded guilty to in a US court, are neither compensable nor preventable by RUCAM.

### 4.2 Simplified AIH score

The simplified AIH score is also a validated diagnostic algorithm directed at the autoimmune aspects of DIAIH (11). It is a widely used scoring system that provides causality gradings ranging from excluded to definite.

### 4.3 ALDEN

ALDEN is a validated algorithm used to assess drug causality in cases of suspected iDILI associated with Stevens–Johnson syndrome and toxic epidermal necrolysis (12). It is a scoring algorithm that provides clear causality gradings.

## 5 Classic iDILI

### 5.1 Basic aspects

Classic or traditional iDILI cases were initially collected to provide the basis for the inauguration of the original RUCAM of 1993 (5) and were submitted subsequently to national DILI registries, whereby all cases received professional RUCAM evaluations by DILI and RUCAM experts (13, 18). Similarly, 81,856 iDILI cases assessed using RUCAM were published worldwide up to mid-2020, outnumbering those evaluated by any other tool in terms of case numbers (19). Per the RUCAM algorithm definition, cases were excluded if increased serum autoimmune parameters were detected (5, 7), thereby classifying all RUCAM-based cases, by tradition, primarily as classic non-immune iDILI (5, 7, 13, 17–19).

The top rankings on drugs implicated in iDILI varied among different countries and regions. An analysis of international RUCAM-based iDILI reports provided a ranking of the top drugs implicated in iDILI (**Table 2**) (10).

**Table 2:** List of drugs most implicated in causing iDILI with verified diagnosis using RUCAM to assess causality.

Drugs and drug classes	RUCAM-based iDILI cases (*n*)
1. Amoxicillin-clavulanate	333
2. Flucloxacillin	130
3. Atorvastatin	50
4 Disulfiram	48
5. Diclofenac	46
6. Simvastatin	41
7. Carbamazepine	38
8. Ibuprofen	37
9. Erythromycin	27
10. Anabolic steroids	26
11. Phenytoin	22
12. Sulfamethoxazole/Trimethoprim	21
13. Isoniazid	19
14. Ticlopidine	19
15. Azathioprine/6-Mercaptopurine	17
16. Contraceptives	17
17. Flutamide	17
18. Halothane	15
19. Nimesulide	13
20. Valproate	13
22. Nitrofurantoin	11
23. Methotrexate	6
24. Rifampicin	7
25. Sulfasalazine	7
26. Pyrazinamide	5
27. Natriumaurothiolate	5
28. Sulindac	5
29. Amiodarone	4
30. Interferon beta	3
31. Propylthiouracil	2
32. Allopurinol	1
33. Hydralazine	1
34. Infliximab	1
35. Interferon alpha/Peginterferon	1
36. Ketoconazole	1

The table was adapted from a previous report published in an open-access journal (1). The RUCAM-based DILI cases represent the total number of cases by drug or drug class and were retrieved from the international literature as specified earlier (20). Abbreviations: iDILI, idiosyncratic drug-induced liver injury; RUCAM, Roussel Uclaf Causality Assessment Method.

### 5.2 Diagnosis

Traditionally, the diagnosis of iDILI was successfully assessed using the original RUCAM in DILI registries (13, 18) and the updated RUCAM in recent reports, with clarification already in the title (19–42). Alternative causes are a problem in iDILI cohorts but are easily recognized by RUCAM (43).

### 5.3 Clinical presentations

The RUCAM-based Spanish DILI registry reported jaundice occurring in 69% of IDILI patients (18), whereas other reports included, in addition to jaundice, abdominal pain, malaise, encephalopathy, bruising, bleeding (44), dark urine, fever, nausea, pruritus, vomiting, and right upper quadrant abdominal pain (45), while asymptomatic clinical courses can be observed concomitantly with low ALT values (46).

### 5.4 Laboratory data

Details of laboratory data can be retrieved from various RUCAM-based reports of the Spanish DILI registry (18): when expressed as multiples of the ULN, ALT was up to 203, ALP up to 32.7, and total bilirubin up to 45.6. Somewhat lower values were reported for RUCAM-based DILI cases included in the Swedish DILI registry (13).

### 5.5 Liver histology

Liver histology results in RUCAM-based DILI cases from the Spanish DILI registry revealed cholestasis (48%) and hepatocellular necrosis (27%) as the predominant features (18). Of note, liver histology data lack specificity and diagnostic value in suspected iDILI cases (7). However, using laboratory data and the RUCAM-based ratio (R) value, hepatocellular injury was found in 58% of cases, cholestatic injury in 20%, and mixed hepatocellular and cholestatic injury in 22% of cases (18).

### 5.6 Treatment and prognosis

Clearly, the use of the offending drug must be stopped as soon as iDILI is suspected (47). If drug cessation fails to improve clinical signs and laboratory results, glucocorticoids (GCs) are commonly used as a first option (38, 47–51); however, whether they are beneficial to patients remains controversial (38, 47, 52). There was no uniform standard for the timing, dosage, and population selection of GCs, which mainly depend on the clinician’s experience (48). In addition, prevailing cohort heterogeneity impaired clear conclusions: highlighted as an international, multicenter, propensity score-matched analysis, the study protocol was suboptimal because one of the included cohorts had been evaluated using a non-validated tool, representing an inappropriate approach; the cohorts ignored the mandatory differentiation of non-immune iDILI from DIAIH, based clearly on autoimmunity as evidenced by positive autoimmune titers in up to 48% cases, and included cases with a merely possible causality grading, supporting the suspicion that cases submitted to the network and registry do not represent prospective studies that would have excluded possible cases *a priori* (49). Patients with cholestatic iDILI were commonly treated with ursodeoxycholic acid (47).

Chronicity was described in 10% of iDILI cases based on RUCAM (18), but, on theoretical grounds, this may well be due to missed alternative causes, pre-existing liver disease, or newly developing flares characterized by autoimmune parameters in the sense of DIAIH. Indeed, many RUCAM-based iDILI cases with a chronic course had persistent alcohol use or hypersensitivity signs such as fever, rash, and/or eosinophilia, associated with normal or increased titers of autoimmune parameters, likely not determined sequentially to confirm or exclude DIAIH rather than iDILI (18). Acute liver failure occurred in 2% of RUCAM-based iDILI cases, liver transplantation was necessary in 2%, and death occurred in 5% of iDILI patients (18).

### 5.7 Molecular pathomechanisms

Immunity and iDILI remain a complex condition, as shown and qualified with ± above (**Table 1**), and calls for refining and completing the theories of pathomechanisms leading to the iDILI (1–3, 53). By its definition, iDILI is traditionally classified as a non-immune and non-autoimmune disorder, a diagnosis established using RUCAM, which removes suspected iDILI cases if autoimmune parameters are detected in the serum of patients (5, 7). Fever, rash and blood eosinophilia are nonspecific features observed in only part of iDILI patients and are not suitable for attributing all iDILI cases to immunity, in line with positive re-exposure data that are restricted to the implicated drug (18). More specifically, the iDILI community is confronted with the proposal that immunity plays a role in part of iDILI cases (1, 3) or even in all cases (2). As GCs are effective in only some patients with iDILI (47–51), an immunological role can be anticipated at best only for those responding to GCs; however, valid percentage data for responders and non-responders are currently not available (1–3, 13, 18, 47–52).

**Figure 1:**
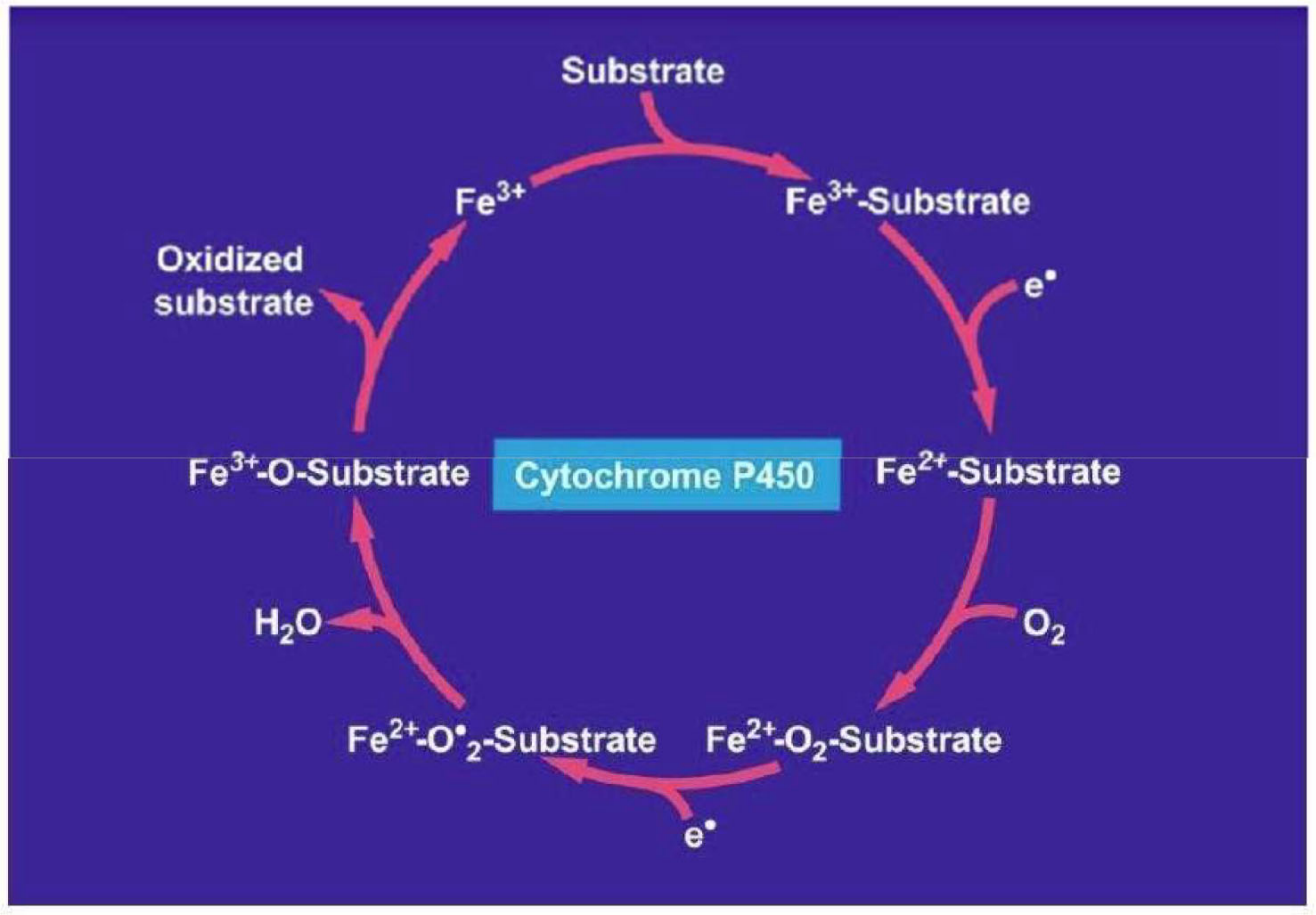
Catalytic cytochrome P450 cycle metabolizing drugs and other exogenous substrates. This figure was derived from a previous report published in an open-access journal (10).

Following hepatic uptake, many drugs are metabolized via CYP pathways (58%) or non-CYP routes, leading to toxic reactive metabolites that cause iDILI with a valid diagnosis verified by RUCAM (3). At least for drugs metabolized via CYP pathways, circumstantial evidence suggests that the sequelae leading to iDILI start with the drug approaching the catalytic CYP cycle and binding to one of its CYP isoforms in its oxidized form (3, 10). Electrons are available from NADPH + H via NADPH–CYP reductase, and the introduction of molecular oxygen leads to the reduced form of CYP, which becomes oxidized again after releasing the oxidized drug. The oxidized CYP is then free again for the next drug to be oxidized. Under normal conditions, this enzymatic process proceeds smoothly, converting a drug substrate into the oxidized drug (**Figure 1**) (10).

In the course of incomplete oxygen splitting during drug metabolism via the catalytic CYP cycle, reactive oxygen species (ROS) are generated, as shown in the lower part of the CYP cycle (**Figure 1**). Part of the ROS is used for carrying out drug metabolism, but if produced in excess by induction of CYP-dependent hepatic microsomal drug-metabolizing enzymes (10), ROS may initiate iDILI, whereby several toxic metabolites are involved (**Table 3**).

**Table 3:** Potentially toxic metabolites of reactive oxygen species (ROS) generated during drug metabolism via the hepatic microsomal cytochrome P450.

Various reactive O_2_-species
Singlet radical ^1^O_2_
Superoxide radical HO_2_
Hydrogen peroxide H_2_O_2_
Hydroxyl radical HO^•^
Alkoxyl radical RO^▪^
Peroxyl radical ROO
Lipid peroxides

These toxic intermediates are injurious to metabolic pathways within hepatocytes and bind to and react with structural phospholipids and proteins as membranous components of subcellular organelles (2, 3, 10, 53). Covalent binding to proteins forms, in turn, neoantigens in some but certainly not all iDILI cases (2). Based on theoretical considerations, drugs or their metabolites can trigger ROS and facilitate hepatocellular oxidative stress. In this setting, iDILI induced by various drugs develops during an adaptive immune reaction involving CD8 cytotoxic T cells in the liver, ultimately leading to hepatocyte cell death (2). The adaptive immune system leading to iDILI requires activation by the innate immune system and is mediated by antigen-presenting cells (APCs). Finally, extracellular damage-associated molecular pattern molecules (DAMPs) released from injured hepatocytes may play a role (1–3, 53). In addition to innate and adaptive lymphocytes, other immune cells are present in the liver, such as infiltrating monocyte-derived macrophages, Kupffer cells, hepatic stellate cells, and liver sinusoidal endothelial cells, closely connected with each other via mediators through processes known as crosstalk, trafficking, or interplay (3, 54, 55). In more detail, the resident innate immune cells in the liver comprise Kupffer cells, dendritic cells, neutrophils, natural killer cells, and natural killer T cells (54, 55). In contrast, CD4 and CD8 T cells represent the adaptative immune system (56). The unique blood supply of the liver also allows for the recruitment of circulating leukocytes upon activation of relevant signaling pathways (55, 57). Consequently, mediators originating in the liver of patients with iDILI could theoretically serve as serum immune biomarkers for diagnosis.

Profiles of serum cytokines, chemokines, and growth factors were analyzed in cases of suspected acute iDILI and described as a model of immune response, differentiating the innate immune system from the adaptive immune system (58). According to this theory and in the context of the innate immune system, immune stimuli derived from damaged tissue initiate NF-κB nuclear translocation and early innate cytokine production (IL-1β, IL-6, TNF-α). Persistence of this early inflammatory state of innate immunity activates adaptive immune processes favoring cellular (T-box transcription factor TBX21, [e.g., T-bet]-dependent/TH1-type: IL-12p70, IFN-γ, IL-2, IL-15, or humoral (GATA3-dependent/TH2-type: IL-4, IL-5, and IL-13) responses. The final study cohort consisted of 32 patients, most of whom showed an innate immune profile, an adaptive immune profile, or combinations thereof. However, eight of 32 (36%) patients displayed a normal immune profile (58). These data can be interpreted as indicating immunological involvement in approximately two-thirds of iDILI patients, whereas approximately one-third of iDILI might have no immune background, supporting earlier contentions that immunology is responsible for most iDILI cases but not for all cases (1). However, there were limitations because the study under consideration was based on cases assessed using the DILIN method (58), a tool known for lacking method validation, arbitrary percentage causality gradings, and subjective evaluations (9). In addition, other iDILI cohorts evaluated using the DILIN method lack case homogeneity because they include cases with overt autoimmune parameters likely attributable to DIAIH rather than iDILI, thereby heavily confounding DILIN results (59, 60). Cytokines, with a preference of serum IL-17, and autoimmune patterns were described in acute liver failure due to iDILI assessed using the disputed DILIN method, a topic contradictory in itself because iDILI is defined without antibody patterns, and if these are present, DIAIH rather than iDILI would be the appropriate term, provided that the simplified AIH score of 2008 have been applied; however, the use of this elementary tool was neglected (59). In general, acute liver failure due to iDILI was broadly reported without any robust causality assessment method (61). Another DILIN-based report describing increased IL-4 levels in clinical iDILI due to volatile anesthetics is critical because the cases are characterized by trifluoroacetyl and CYP2E1 antibodies, which makes them as DIAIH rather than iDILI (60).

## 6 DIAIH

### 6.1 Basic aspects

Case numbers of DIAIH rank second after those of classic iDILI (19). DIAIH consists of two parts: one reflects the iDILI component and the other reflects the AIH component (8). While the iDILI component has well been analyzed above in relation to classic iDILI, AIH features requires thorough discussion. In the past, DIAIH evaluation was largely neglected and often not differentiated from other immune iDILI types (8).

### 6.2 Diagnosis

The use of the updated RUCAM (7) and the simplified AIH score (11) is mandatory to establish the diagnosis of DIAIH (8). Because the simplified AIH score requires a liver histology obtained via liver biopsy, an invasive procedure, the simplified AIH score should be used after the updated RUCAM has confirmed the iDILI diagnosis to avoid unnecessary liver biopsy in cases of non-verified iDILI components (8). By applying both diagnostic algorithms, specific drugs are listed as causing DIAIH with verified diagnosis (**Table 4**) (62–73).

**Table 4:** Drugs and drug groups implicated in published DIAIH cases with diagnoses verified using the validated causality algorithms of both the RUCAM and the simplified AIH score.

Drugs and drug groups	Cases (*n*)	References
Adalimumab	1 1	Martínez-Casas, 2018 (62) Chung, 2024 (63)
Allopurinol	1	Chung, 2024 (63)
Amitriptyline	1	Weber, 2019 (64)
Amoxicillin-Clavulanate	2	García-Cortés, 2023 (65)
Amoxicillin-Clavulanate + Ceftriaxone	3	Licata, 2014 (66)
Amoxicillin + Erythromycin	1	Chung, 2024 (63)
Amoxicillin + Metronidazole	1	Chung, 2024 (63)
Anabolic steroid	1	Chung, 2024 (63)
Atorvastatin	2 2 2 1 1	Yeong, 2016 (67) Weber, 2019 (64) García-Cortés, 2023 (65) Tan, 2022 (68) Tse, 2023 (69)
Candesartan	1	Hassoun, 2023 (70)
Cephalexin + Amoxicillin	1	Chung, 2024 (63)
Ciprofloxacin	1 1	García-Cortés, 2023 (65) Chung, 2024 (63)
Cyproterone acetate	2	García-Cortés, 2023 (65)
Dabigatran	1	Weber, 2019 (64)
Dexketoprofen	1	García-Cortés, 2023 (65)
Diclofenac	1 2 3	Yeong, 2016 (67) Martínez-Casas, 2018 (62) Weber, 2019 (64)
Ebrotidine	1	García-Cortés, 2023 (65)
Efalizumab	1	García-Cortés, 2023 (65)
Enalapril maleate	1	Hassoun, 2023 (70)
Etanercept	1	Valgeirsson, 2019 (71)
Ezetimibe	1	García-Cortés, 2023 (65)
Fluvastatin	4	García-Cortés, 2023 (65)
Fosfomycin	1	Hassoun, 2023 (70)
Ibandronate	1	Hassoun, 2023 (70)
Ibuprofen	5 1	Hassoun, 2023 (70) García-Cortés, 2023 (65)
Imatinib	1 1 1	Björnsson, 2017 (72) Weber, 2019 (64) Valgeirsson, 2019 (71)
Infliximab	8 7 1 1 1 1	Björnsson, 2017 (72) Valgeirsson, 2019 (71) Chung, 2024 (63) García-Cortés, 2023 (65) Weber, 2019 (64)
	1	Weber, 2019 (64)
Irbesartan	1	García-Cortés, 2023 (65)
Isotretinoin	1	García-Cortés, 2023 (65)
Lansoprazole	1	Chung, 2024 (63)
Lymecycline	2	Chung, 2024 (63)
Mefenamic acid	1	Hassoun, 2023 (70)
Menotropin	1	Alqrinawi, 2019 (73)
Metamizole	3	Weber, 2019 (64)
Methocarbamol	1	Weber, 2019 (64)
Nimesulide + Ketoprofen	6	Licata, 2014 (66)
Minocycline	4 4 1	García-Cortés, 2023 (65) Chung, 2024 (63) Weber, 2019 (64)
NSAIDs + Antibiotics	1	Chung, 2024 (63)
Natalizumab	1	Valgeirsson, 2019 (71)
Nitrofurantoin	8 7 5 4 3 1	Martínez-Casas, 2018 (62) Chung, 2024 (63) García-Cortés, 2023 (65) Yeong, 2016 (67) Björnsson, 2017 (72) Hassoun, 2023 (70)
Olmesartan	1	Hassoun, 2023 (70)
Orlistat	1	García-Cortés, 2023 (65)
Pembrolizumab	1	Weber, 2019 (64)
Propylthiouracil	1	Martínez-Casas, 2018 (62)
Rivaroxaban	1	Weber, 2019 (64)
Rosuvastatin	1	García-Cortés, 2023 (65)
Simvastatin	1 1	Yeong, 2016 (67) García-Cortés, 2023 (65)
Sorafenib	1	Tan, 2022 (68)
Trazodone	2	Hassoun, 2023 (70)
Valsartan	1	Hassoun, 2023 (70)

Compilation of selected drugs implicated in causing DIAIH, whereby RUCAM assessing the DILI component, commonly and correctly refers to the original version (5) or its updated version (7), and the AIH component is commonly evaluated using the simplified criteria of the AIH score (11) or, rarely, one of its modifications. The table was modified from a previous report published in an open-access journal (2). Abbreviations: DIAIH, Drug-Induced Autoimmune Hepatitis; NSAIDs, Nonsteroidal Anti-Inflammatory Drugs; RUCAM, Roussel Uclaf Causality Assessment Method.

Twenty DIAIH reports were initially analyzed (8), of which 12 of 20 reports (60%) were correctly assessed using RUCAM and the simplified AIH score (**Table 3**). This shows that the majority of initially suspected DIAIH cases ultimately received a firm diagnosis, a fairly good result in the face of cohort heterogeneity (8). Conversely, reports were published that had been partially or not at all correctly assessed using the mandatory algorithms (5, 7, 11), rendering the results reported therein questionable (**Table 5**) (74–81).

**Table 5:** Selected drugs implicated in suspected but not verified DIAIH with unverified diagnosis.

Drugs	Cases (*n*)	RUCAM causality algorithm used	Simplified criteria of AIH score used	DIAIH diagnosis verified by both the RUCAM and the simplified AIH score	References
Adalimumab	1 1	YES NO	NO YES	NO NO	Ghabril, 2013 (74) Rodrigues, 2015 (75)
Atorvastatin	1	YES	NO	NO	Khan, 2020 (76)
Cephalexin	1	NO	YES	NO	Björnsson, 2010 (77)
Etanercept	2	YES	NO	NO	Ghabril, 2013 (74)
Hydralazine	7	NO	NO	NO	de Boer, 2017 (78)
Infliximab	25 8 3	YES NO YES	NO YES NO	NO NO NO	Björnsson, 2022 (79) Rodrigues, 2015 (75) Ghabril, 2013 (74)
Methyldopa	10	NO	NO	NO	de Boer, 2017 (78)
Minocycline	19 10 1	NO NO NO		NO NO NO	de Boer, 2017 (78) Björnsson, 2010 (77) Harmon, 2018 (80)
Nitrofurantoin	24 10	NO NO	NO YES	NO NO	de Boer, 2017 (78) Björnsson, 2010 (77)
Pirfenidone	1	YES	NO	NO	Fortunati, 2024 (81)
Prometrium	1	NO	YES	NO	Bjornsson, 2010 (77)

Compilation of suspected drugs implicated in causing DIAIH but without complete diagnostic causality verification using validated methods. For some patients, the DILI component of DIAH was causally evaluated solely by the original RUCAM (5) or the updated RUCAM (7), while other cases were submitted merely to the assessment of the AIH part by the simplified criteria of the AIH score (11) or rarely by one of its modifications. A group of DIAIH patients was evaluated using none of these methods. The table was modified and derived from a previous report published in an open-access journal (8). Abbreviations: DIAIH, Drug-Induced Autoimmune Hepatitis; NSAIDs, Nonsteroidal Anti-Inflammatory Drugs; RUCAM, Roussel Uclaf Causality Assessment Method.

From the initially analyzed 20 studies of suspected DIAIH (8), in four of 20 reports (20%) only RUCAM was used (74, 76, 79, 81), and in two of 20 reports (10%) applied only the simplified AIH score (75, 77) with the consequence that these evaluations did not allow for a valid DIAIH diagnosis in these six reports (**Table 5**). It was striking that two of 20 reports (10%) were presented without either of the two causality algorithms (78, 80) and therefore contained inconclusive feature data of DIAIH, lacking scientific or clinical value. Due to these methodological shortcomings, several reports should be excluded from further DIAIH characterization (74–81).

### 6.3 Clinical manifestations

#### 6.3.1 Issue of non-drug causes

Expectations to establish a correct DIAIH diagnosis are high because the risk of missed diagnoses is high if alternative causes are not correctly excluded (64, 67, 71), a problem also connected to iDILI (43). More specifically, non-drug competing diagnoses have been detected in the course of DIAIH case evaluation (64, 67, 71). As an example, a careful DIAIH study showed alternative causes in 35.5% of patients, with AIH being the most common (10.5%), followed by cholangitis and cholelithiasis (5.2%), hepatitis E virus (4.2%), alcohol (3.5%), cardiac failure (2.8%), secondary sclerosing cholangitis (2.1%), non-alcoholic steatohepatitis, now known as metabolic dysfunction-associated steatohepatitis (1.7%), other autoimmune diseases (1.7%), metabolic disorders such as Wilson disease and hemochromatosis (1.4%), primary biliary cholangitis (1.4%), primary sclerosing cholangitis (1.1%), non-viral infections like abscesses and echinococcosis (1.1%), hepatitis A virus (0.7%), human herpes virus (0.7%), Epstein–Barr virus (0.7%), cytomegalovirus (0.4%), malignant infiltration (0.4%), and others (1.1%) (64). Overall, the most frequent alternative was AIH (10.5%), followed by cholangitis/cholelithiasis (5.2%), hepatitis E virus (4.2%), alcohol (3.5%), cardiac failure (2.8%), secondary sclerosing cholangitis (2.1%), and metabolic dysfunction-associated steatohepatitis (1.7%) (64, 67, 71).

#### 6.3.2 Causality gradings

Describing the clinical specifics of DIAIH requires a careful selection of reported cohorts that include cases of patients with an established diagnosis using both the RUCAM and the simplified AIH score to ensure robust causality for the implicated drug (**Table 3**). For these reasons, cases were selected from well-evaluated DIAIH reports that included 49 different drugs, drug groups, and drug combinations in a total of 25 DIAIH cases (**Table 3**). In these cases, RUCAM scores were up to 10 (62, 64) or 6–8 (67), and AIH scores ranged up to 14 (62, 64) or 10–17 (67). In other words, for all evaluated cases, a causality grading of probable or highly probable was attributed.

#### 6.3.3 Symptoms and clinical specifics

Many RUCAM-based DIAIH cases were included in study cohorts (**Table 3**). However, the aim of these reports was not necessarily directed toward the symptoms and clinical presentation of patients experiencing DIAIH. A better approach is likely the search for clinical details published in single-patient reports. As an example, there is a report of a patient who was diagnosed with DIAIH following therapy with menotrophin and developed pale stool and dark urine (73). Such symptoms are considered nonspecific features similar to various other hepato-biliary disorders that may confound the DIAIH diagnosis. This uncertainty calls for a robust causality assessment using RUCAM, which helps identify and exclude alternative causes. Scleral icterus associated with an otherwise unremarkable clinical presentation was described in another patient with DIAIH due to sorafenib (68), and jaundice was reported in another DIAIH patient observed following treatment with atorvastatin (69). In another report of 28 patients with DIAIH due to various drugs, jaundice/pruritus was observed in 20 cases, fatigue/malaise in seven cases, abdominal pain in three cases, and arthralgia in one case (63). Jaundice at onset was mentioned in eight of 12 patients diagnosed with DIAIH (66).

### 6.4 Laboratory data

Increased titers of serum autoimmune parameters are fundamental elements of the simplified AIH score (11) and help establish the diagnosis of DIAIH (8). These parameters include IgG, ANA, ASMA, and SLA, but they were often not specified. ANA is the most frequent autoimmune parameter, with positive titers found in 77.3% of DIAIH patients (64). Laboratory data and autoimmune parameters are listed as reported for a few patients with DIAIH caused by selected drugs (**Table 6**) (62–73).

**Table 6:** ALT and ALP values as well as autoimmune parameters as described in cases of DIAIH caused by specific drugs and drug groups.

Drugs	Cases (*n*)	ALT (U/L)	ALP (U/L)	Autoimmune parameters	References
Adalimumab	1	562	NR	ANA	Martínez-Casas, 2018 (62)
Amitriptyline	1	NR	NR	Not specified	Weber, 2019 (64)
Amoxicillin- Clavulanate	2	NR	NR	Not specified	García-Cortés, 2023 (65)
Amoxicillin- Clavulanate + Ceftriaxone	3	NR	NR	Not specified	Licata, 2014 (66)
Amoxicillin + Erythromycin	1	NR	NR	Not specified	Chung, 2024 (63)
Amoxicillin + Metronidazole	1	NR	NR	Not specified	Chung, 2024 (63)
Anabolic steroid	1	NR	NR	Not specified	Chung, 2024 (63)
Atorvastatin	2 2 2 1 1	721 NR NR 696 385	NR NR NR 107 163	ANA, ASMA Not specified Not specified Unremarkable ANA	Yeong, 2016 (67) Weber, 2019 (64) García-Cortés, 2023 (65) Tan, 2022 (68) Tse, 2023 (69)
Candesartan	1	NR	NR	Not specified	Hassoun, 2023 (70)
Cefalexin + Amoxicillin	1	NR	NR	Not specified	Chung, 2024 (63)
Ciprofloxacin	1	NR	NR	Not specified	García-Cortés, 2023 (65)
Cyproterone acetate	2	NR	NR	Not specified	García-Cortés, 2023 (65)
Dabigatran	1	NR	NR	Not specified	Weber, 2019 (64)
Dexketoprofen	1	NR	NR	Not specified	García-Cortés, 2023 (65)
Diclofenac	1 2 3	3489 1491 NR	NR NR NR	ANA, ASMA ANA, ASMA, SLA Not specified	Yeong, 2016 (67) Martínez-Casas, 2018 (62) Weber, 2019 (64)
Ebrotidine	1	NR	NR	Not specified	García-Cortés, 2023 (65)
Efalizumab	1	NR	NR	Not specified	García-Cortés, 2023 (65)
Ezetimibe	1	NR	NR	Not specified	García-Cortés, 2023 (65)
Enalapril maleate	1	NR	NR	Not specified	Hassoun, 2023 (70)
Fluvastatin	4	NR	NR	Not specified	García-Cortés, 2023 (65)
Fosfomycin	1	NR	NR	Not specified	Hassoun, 2023 (70)
Ibandronate	1	NR	NR	Not specified	Hassoun, 2023 (70)
Ibuprofen	5 1	NR NR	NR NR	Not specified Not specified	Hassoun, 2023 (70) García-Cortés, 2023 (65)
Imatinib	1 1	1212 NR	205 NR	ANA Not specified	Björnsson, 2017 (72) Weber, 2019 (64)
Infliximab	10 8 1 1	1658 NR NR NR	493 NR NR NR	ANA ASMA Not specified Not specified	Björnsson, 2017 (72) Valgeirsson, 2019 (71) García-Cortés, 2023 (65) Weber, 2019 (64)
Interferon beta	1	NR	NR	Not specified	
Irbesartan	1	NR	NR	Not specified	García-Cortés, 2023 (65)
Isotretionin	1	NR	NR	Not specified	García-Cortés, 2023 (65)
Lansoprazole	1	NR	NR	Not specified	Chung, 2014 (63)
Lymecycline	2	NR	NR	Not specified	Chung, 2024 (63)
Mefenamic acid	1	NR	NR	Not specified	Hassoun, 2023 (70)
Menotropin	1	504	366	ANA	Alqrinawi, 2019 (73)
Metamizole	3	NR	NR	Not specified	Weber, 2019 (64)
Methocarbamol	1	NR	NR	Not specified	Weber, 2019 (64)
Minocycline	4 4 1	NR NR NR	NR NR NR	Not specified Not specified Not specified	García-Cortés, 2023 (65) Chung, 2024 (63) Weber, 2019 (64)
Nimesulide + Ketoprofen	6	NR	NR	Not specified	Licata, 2014 (66)
Nitrofurantoin	8 7 5 4 3 1	2059 NR NR 587 1974 NR	NR NR NR NR 204 NR	ANA, ASMA, Not specified Not specified ANA, ASMA ANA Not specified	Martínez-Casas, 2018 (62) Chung, 2024 (63) García-Cortés, 2023 (65) Yeong, 2016 (67) Björnsson, 2017 (72) Hassoun, 2023 (70)
NSAIDs + Antibiotics	1	NR	NR	Not specified	Chung, 2024 (63)
Olmesartan	1	NR	NR	Not specified	Hassoun, 2023 (70)
Orlistat	1	NR	NR	Not specified	García-Cortés, 2023 (65)
Pembrolizumab	1	NR	NR	Not specified	Weber, 2019 (64)
Propylthiouracil	1	754	NR	ANA	Martínez-Casas, 2018 (62)
Rivaroxaban	1	NR	NR	Not specified	Weber, 2019 (64)
Rivaroxaban	1	NR	NR	Not specified	Weber, 2019 (64)
Rosuvastatin	1	NR	NR	Not specified	García-Cortés, 2023 (65)
Simvastatin	1 1	1245 NR	NR NR	ANA, ASMA Not specified	Yeong, 2016 (67) García-Cortés, 2023 (65)
Sorafenib	1	1004	190	Unremarkable	Tan, 2022 (68)
Trazodone	2	NR	NR	Not specified	Hassoun, 2023 (70)
Valsartan	1	NR	NR	Not specified	Hassoun, 2023 (70)

Compilation of selected drugs and drug groups implicated in causing DIAIH, whereby RUCAM assessing the DILI part commonly and correctly stands for the original version (5) or its updated version (7), and the AIH part is commonly evaluated by the simplified criteria of the AIH score (11) or rarely by one of its modifications. The list was derived from a previous report published in an open access journal (8). Abbreviations: ANA, Anti-Nuclear Antibodies; ASMA, Anti-Smooth Muscle Antibodies; DIAIH, Drug-Induced Autoimmune Hepatitis; NR, Not Reported; NSAIDs, Nonsteroidal Anti-Inflammatory Drugs; RUCAM, Roussel Uclaf Causality Assessment Method; SLA, Soluble Liver Antigen antibodies.

Current knowledge on autoimmune parameters in DIAIH is scattered (**Table 4**), particularly because closely sequential data from the onset of DIAIH are lacking. It is unclear whether autoimmune parameters become detectable concomitantly with increased ALT values or only later, suggesting that the autoimmune process may emerge with delay.

### 6.5 Liver histology

Liver histological data are among the most important diagnostic cornerstones of the simplified AIH score for establishing the diagnosis of DIAIH (15), but they were rarely included in the reports (**Table 3**). Detailed histological lesions of DIAIH were available from a few published reports, all assessed by RUCAM and the simplified AIH score (63, 65, 66, 68–70): portal and lobular inflammation with lobular disarray (68), lobular hepatitis (63, 70), chronic inflammation (68), mixed periportal necroinflammatory infiltrate with increased plasma cells and ductular reaction (69), multiacinar parenchymal loss with ductular reactions and inflammatory infiltrates (63), confluent necrosis (70), entrapped hepatocytes with rosette architecture (63, 70), rosettes (65), ballooned hepatocytes (65), hepatocytes with rosetting Councilman bodies and hepatocyte drop-outs (68), plasma cell infiltrates (63, 68), lymphoplasmacytic infiltrates (65, 66, 68, 70), monocytic infiltration (65), features of chronic active hepatitis (63), interface hepatitis (65, 68, 70), portal inflammation (66), portal tract expansion with inflammatory infiltrate associated with interface hepatitis and plasma cell aggregates (63), portal tracts with aggregated eosinophils (63), rare eosinophilia (65, 68), fibrosis (65), and mild steatosis (66).

### 6.6 Treatment and prognosis

Therapy starts with cessation of the drug as soon as DIAIH is suspected (8). If cessation lacks therapeutic efficacy, immunosuppressive agents are needed (**Table 7**).

**Table 7:** Selected drugs implicated in DIAIH with treatment response of cessation of the suspect drug or after immunosuppressive therapy.

Drugs	Cases (*n*)	Response of drug stop or therapy	References
Adalimumab	1 1	CR with PRED/AZA CR with cessation of the culprit drug	Chung, 2024 (62) Weber, 2019 (63)
Allopurinol	1	CR with IS	Weber, 2019 (63)
Amoxicillin + Erythromycin	1	CR with IS	Weber, 2019 (63)
Amoxicillin + Metronidazole	1	CR with IS	Weber, 2019 (63)
Anabolic steroid	1	CR with IS	Weber, 2019 (63)
Atorvastatin	1 2	CR with PRED CR with cessation of the culprit drug	Tse, 2023 (68) Hassoun, 2023 (69)
Cefalexin + Amoxicillin	1	CR with IS	Weber, 2019 (63)
Ciprofloxacin	1	CR with IS	Weber, 2019 (63)
Diclofenac Diclofenac + Ibuprofen	1 1	CR with PRED/AZA IR with PRED/AZAI TAC/UCDA	Chung, 2024 (62) Weber, 2019 (63)
lnfliximab	1 2	CR with IS CR with cessation of the culprit drug	Weber, 2019 (63) Weber, 2019 (63)
Lansoprazole	1	CR with IS	Weber, 2019 (63)
Menotropin	1	CR with PRED/AZA	Ghabril, 2013 (73)
Minocycline	1	CR with IS	Weber, 2019 (63)
NSAIDs + Antibiotics	1	CR with IS	Weber, 2019 (63)
Nitrofurantoin	8 7	CR with PRED/AZA CR with IS	Chung, 2024 (62) Weber, 2019 (63)
Propylthiouracil	1	CR with PRED/AZA	Chung, 2024 (62)
Sorafenib	1	CR with cessation of the culprit drug	Tse, 2023 (68)

Compilation of selected drugs implicated in causing DIAIH, with specification of therapeutic modalities and their efficacy. The DILI component of DIAIH was assessed by the original RUCAM (5) or its updated version (7), while the AIH component was evaluated using the simplified AIH score (11) or, rarely, one of its modifications. The table was modified from a previous report published in an open-access journal (8). Abbreviations: AZA, azathioprine; CR, complete response; DIAIH, drug-induced autoimmune hepatitis; IR, incomplete response; IS, immunosuppressants, not further specified; NSAIDs, nonsteroidal anti-Inflammatory drugs; PRED, prednisolone; RUCAM, Roussel Uclaf Causality Assessment Method; TAC, tacrolimus; UDCA, ursodeoxycholic acid.

Notably, a few DIAIH patients experienced complete remission following cessation of the causative drug (**Table 7**), a phenomenon also observed in other types of iDILI. Remission following drug cessation alone was found in cases of DIAIH due to adalimumab (62), atorvastatin (69), infliximab (63), and sorafenib (68). Cessation obviously helps not only the acute liver injury component but, surprisingly, also the AIH component. Other patients with DIAIH commonly received after cessation of the suspect drug, induction therapy with immunomodulators such as prednisolone (PRED) (62) or methylprednisolone (64), combined with azathioprine (AZA) (62), while AZA alone was used as maintenance therapy (62). Treatment with tacrolimus and ursodeoxycholic acid (UDCA) was rare (63). The time interval until initiation of corticosteroid therapy was reported as a mean of 19.5 days, with a range of 7 to 195 days (64).

The response to induction and maintenance treatment was commonly favorable (62–64), whereas the frequency of acute liver failure requiring liver transplantation varied among DIAIH patients, occurring in 4.6% (54) and 14.3% (63) of cases or not being reported (62). For 7.1% of DIAIH patients, the outcome was poor, leading to death (63).

### 6.7 Molecular pathomechanisms

Briefly summarized, reactive metabolites generated from the hepatic metabolism of drugs bind to cellular proteins such as components of CYP (82), which are then recognized as neoantigens by a heightened immunological response, leading to the AIH component of DIAIH (68, 82) as a result of misdirected immune response (68).

#### 6.7.1 DILI part of DIAIH

Molecular and mechanistic steps leading to the DILI component of DIAIH are likely similar to those described above for iDILI, with a focus on the roles of CYP-dependent and non-CYP-dependent pathways, ROS, immune and non-immune systems, innate and adaptive immune reactions, hepatic immune cells, and crosstalk among mediators.

#### 6.7.2 Autoimmune part of DIAIH

Evaluating the immunological steps underlying the autoimmune features of DIAIH requires an examination of the mechanistic details of idiopathic AIH, including its genetics (82–85). Genetic predisposition plays a pivotal role in the development of AIH, as evidenced by a substantial association with specific HLA types, in particular HLA-DRB1*0301 (83). However, this genetic condition cannot be transferred as a trigger to the AIH component of DIAIH, for which serum HLA data are not available among the large series of DIAIH cases with a robust diagnosis of DIAIH (**Table 4**) (62–73). However, it has been suggested that DIAIH is related to genetic polymorphisms, a claim not substantiated by appropriate studies and in contradiction to the absence of a specific HLA haplotype (85), according to published experimental and clinical reports (62, 86, 87). Thus, while DIAIH develops without genetic predisposition and in the absence of HLA associations, AIH is genetically determined, with HLA acting as a prominent trigger. The involvement of T cells in AIH (83) is shared with iDILI (2) and therefore likely also with the DILI component of DIAIH. As T cell-driven diseases, AIH is triggered by HLA (83), and the DILI component of DIAIH is likely initiated by toxic reactive metabolites of the drug, conditions that result in newly emerging serum autoimmune parameters in high titers in both AIH (83–85) and DIAIH (**Table 6**) (62–73).

#### 6.7.3 Encouraging pathophysiological insights from the two injurious flares in DIAIH

New and encouraging insights into the pathophysiology of DIAIH can be derived from DIAIH case reports (74, 88), describing the two-flare phenomenon consisting of a first injurious flare classified as the acute liver injury component of DIAIH, followed by a second injurious autoimmune flare with progression to clinical autoimmunity (89). The first and most striking DIAH case was associated with the use of the smoking cessation agent varenicline (88), and the second DIAIH case was attributed to treatment with intravenous infliximab for ankylosing spondylitis (74).

##### 6.7.3.1 DIAIH by varenicline

The varenicline DIAIH case from Japan was characterized by an excellent clinical and diagnostic analysis, with the diagnosis ascertained by a modified RUCAM and the simplified AIH score, and provided important clinical details (88): (1) liver injury started 5 days after daily treatment with varenicline at recommended doses, with increased serum ALT values of 886 U/L and ALP of 419 U/L, as well as normal total bilirubin values of 1.3 mg/dL, in the absence of viral or autoimmune responses; withdrawal of varenicline and treatment with ursodeoxycholic acid immediately lowered the liver enzyme levels, as shown in **Figure 1**, with ALT values of approximately 80 U/L and ALP values of approximately 200 U/L; and (2) surprisingly, the patient was readmitted to the hospital four weeks after the previous hospitalization because of increased serum aminotransferase levels detected during a follow-up examination. Although the physical evaluation was again unremarkable, ALT was 588 U/L and total bilirubin of 0.7 mg/dL, but serum ANA titers had become positive and signified newly emerged AIH features under conditions of unchanged serum IgG levels (88). Notably, the scientific advisors of the NIH financed US LiverTox database included the varenicline case as a superficial narrative without their own analysis of the important clinical findings, ignored the causality assessment data, and, even worse, failed to classify the case as DIAIH, thereby disregarding not only the significance of the finding but also new developments that had emerged in this specialized field of DIAIH (90).

At the pathophysiological level, varenicline can cause not only DIAIH (88) but also iDILI (88, 89); it belongs to the minor group of drugs (41.7%) that are metabolized via non-CYP pathways (88, 89, 91) and represents a drug known to cause iDILI with a verified diagnosis by RUCAM (3). Among the drugs not metabolized by CYP isoforms are, in addition to varenicline (88, 89, 91), allopurinol (92), amoxicillin–clavulanate (93), azathioprine/6-mercaptopurine (94), busulfan (95), dantrolene (96), didanosine (97), floxuridine (98), hydralazine (99), infliximab (100), interferon alpha/peginterferon (101), interferon beta (102), ketoconazole (103), methotrexate (104), minocycline (105), natriumaurothiolate (13), nitrofurantoin (106), pyrazinamide (107), rifampicin (108), sulfasalazine (109), and thioguanine (110), some of which can also cause DIAIH (**Table 4**). Upon CYP-independent degradation, varenicline or its ROS-mediated toxic radicals will initiate the acute liver injury attack as the first flare of DIAIH through process likely similar to those observed in iDILI caused by CYP-dependent drugs, as proposed above (**Figure 1**, **Table 3**) and described in detail previously (3, 89). For the first flare in the current varenicline case, a role for immune or autoimmune events is far from evidence; instead, the autoimmune process developed long after the first flare in the varenicline patient under consideration (88). The long interval can be explained by the high systemic availability of varenicline due to its slow degradation (89, 91, 111–115). Presumably, over the long term, neoantigens are gradually formed in hepatocytes through covalent binding of the modified parent drug or reactive varenicline metabolites and ROS to cellular proteins; covalent binding occurs at membrane constituents of hepatic mitochondria and the endoplasmic reticulum, corresponding to the microsomal fraction described by biochemists (89). Neoantigens are responsible for the antibody response, and autoimmune reactions eventually manifest as a second flare of DIAIH with detection of newly emerging autoimmune parameters in the blood, as in the patient following varenicline treatment (88, 89). However, the individual neoantigen(s) responsible for the second flare of DIAIH induced by varenicline remain undetermined (89). While AIH is genetically governed by HLA (83), this does not apply to the autoimmune response of the second flare (88). ALT levels were higher during the first flare compared with the second flare, indicating that the autoimmune injurious attack is weaker.

##### 6.7.3.2 DIAIH by infliximab

In another patient, treatment with infliximab, a TNF-α antagonist, caused DIAIH with two flares (74) with findings similar to those reported in the varenicline-associated DIAIH patient from Japan (88). The two DIAIH flares were described in a male patient from the US with ankylosing spondylitis following treatment with intravenous infliximab, with an ALT level of 1,270 U/L at the first flare (74). After drug cessation, ALT fell to 198 U/L within two months after the last infusion but subsequently rose to 1,167 U/L, indicating a less severe injurious attack attributable to autoimmunity. Initially, the serum ANA titer was negative but became positive one month later during the second flare. Prednisone was started, and serum ALT normalized within 2 months, accompanied by serum ANA titers that reverted to negative. Using RUCAM, a possible causal relationship with infliximab was established, whereas attempts to apply the DILIN method remained highly questionable (74), as this tool lacks internal and external validation (9). Difficult to reconcile was the fact that the simplified AIH score of 2008 (11) was not used; therefore, the case should be categorized as possible DIAIH. At the time of publication of this infliximab report in 2013, the 11 DILIN authors were apparently not yet familiar with DIAIH and the requirements for establishing a robust diagnosis (74), including the use of the simplified AIH score (11). From a pathophysiological perspective, it is noteworthy that infliximab, as a monoclonal antibody and therefore a protein (100), is not metabolized by hepatic CYPs or other oxidoreductases but most likely by nonspecific proteases (89, 116). The metabolic disposition of infliximab is slow, and there is no evidence that infliximab itself undergoes significant metabolic transformation in the liver; the specific breakdown products are unknown and are possibly eliminated via the reticuloendothelial system, primarily through macrophages and other immune cells (89, 117, 118). Serum antidrug antibodies to infliximab in patients receiving infliximab therapy are signs of active immune reactions (118–121). Overall, however, substantial information gaps exist regarding possible specific infliximab metabolites and how they, together with infliximab as the parent drug, interact with the innate and adaptive immune systems to eventually trigger the autoimmune events leading to the second flare of DIAIH following the intravenous administration of infliximab (74, 89).

## 7 Idiosyncratic drug-induced anti-CYP autoimmune hepatitis

### 7.1 Basic aspects

As a reminder, among the drugs implicated in causing iDILI, 58.3% are metabolized by CYP isoforms, with CYP-mediated drug metabolism being closely associated with RUCAM-based iDILI (3), and autoimmune reactions related to some CYP isoforms, manifested as anti-CYP antibodies, are to be expected (4, 10). This applies to a few drugs metabolized by the CYP isoforms CYP1A2, CYP2C9, CYP2E1, and CYP3A (10). This allows for the classification of idiosyncratic drug-induced anti-CYP autoimmune hepatitis as a separate type of iDILI (**Table 1**). However, not all cases of suspected idiosyncratic drug-induced anti-CYP autoimmune hepatitis were assessed for causality using RUCAM (4, 10).

Serum anti-CYP antibodies are the hallmark of cases classified as drug-induced anti-CYP autoimmune hepatitis, with the diagnosis verified by RUCAM and causality established for sevoflurane and desflurane (10). Among the few other suspected drugs or drug groups known to cause this type of iDILI are, in alphabetical order, antiepileptic drugs, dihydralazine, halothane, isoflurane, isoniazid, and tienilic acid; however, none of their cases benefited from causality assessment using RUCAM (**Table 8**) (10, 15, 122–130).

**Table 8:** Serum anti-CYP antibodies in patients with idiosyncratic drug-induced RUCAM-based DILI following use of volatile anesthetics.

Drug	Type of antibody	RUCAM used	Details of idiosyncratic drug-induced anti-CYP autoimmune hepatitis	References
Antiepileptic drugs	Anti-CYP3A	NO	Narratives with disputable results due to missing exclusion of any alternative causes and lacking causality assessment by RUCAM.	Meunier, 2019 (122) Obermayer- Straub, 2000 (123)
Dihydralazine	Anti-CYP1A2	NO	Narratives with disputable results due to missing exclusion of any alternative causes and lacking causality assessment by RUCAM.	Meunier, 2019 (122) Obermayer- Straub, 2000 (123)
Halothane	Anti-CYP2E1	NO	Narratives with disputable results due to missing exclusion of any alternative causes and lacking causality assessment by RUCAM. Halothane is now rarely used.	Jee, 2021 (126) Njoku, 2002 (127) Njoku, 2006 (128) Bourdi, 1996 (129) Kenna, 1987 (130)
Isoflurane	Anti-CYP2E1	NO	Narrative with disputable results due to mssing exclusion of any alternative causes and lacking causality assessment by RUCAM.	Njoku,2002 (127)
Isoniazid	Anti-CYP2C9 Anti-CYP2E1 Anti-CYP3A4 Anti-INH	NO	Narrative comprising interesting, suspected cases but lacking assessment by RUCAM. In the INH cohort,11 patients had anti-CYP2E1 anti-bodies,14 had anti-bodies against CYP2E1 modified by INH, 14 had anti-CYP3A4 anti-bodies, and 10 had anti-CYP2C9 anti-bodies. Anti-INH anti-bodies were present in 8 patients.	Metushi, 2014 (124)
Sevoflurane	Anti-CYP2E1 Anti-TFA	YES	A highly probable RUCAM-based causality grading was determined in 4 patients with positive titers of serum anti-CYP2E1 anti-bodies and anti-TFA anti-bodies following application of sevoflurane, a volatile anesthetic. These cases are to be classified as typical idiosyncratic drug-induced anti-CYP autoimmune hepatitis. Proper exclusion of alternative causes.	Nicoll, 2012 (125)
Sevoflurane + Desflurane	Anti-CYP2E1 Anti-TFA	YES	A highly probable RUCAM-based causality grading was determined for 3 patients and a probable causality for 5 patients, in most of these sevoflurane was applied alone, with a few patients receiving desflurane alone or combined with sevoflurane, but increased serum titers of anti-CYP2E1 anti-bodies and anti-TFA anti-bodies were found in only a few patients of this prospective study. In these few patients, the final diagnosis of idiosyncratic drug-induced anti-CYP autoimmune hepatitis was confirmed. Special care focused on considering alternative causes like infections, trauma, sepsis, hypotension, and DILI by APAP or anti-biotics	Bishop, 2019 (15)
Tienilic acid	Anti-CYP2C9	NO	Narrative with disputable results due to missing exclusion of alternative causes and lacking causality assessment by RUCAM.	Meunier, 2019 (122)

The table was modified from and derived from a previous open-access article (10). Abbreviations: APAP, N-acetyl-para-aminophenol, better known as acetaminophen or paracetamol; CYP, cytochrome P450; DILI, drug-induced liver injury; RUCAM, Roussel Uclaf Causality Assessment Method; TFA, trifluoroacetyl.

Idiosyncratic drug-induced anti-CYP autoimmune hepatitis has been described for various drugs as causative compounds, but not all cases were assessed for causality using RUCAM, making the published data fragile (**Table 8**) because it is well known that many cases of iDILI were not caused by drugs but were attributable to alternative, nondrug causes (43). However, only sevoflurane cases received a comprehensive assessment and provided a good overview of this special type of iDILI (15, 125).

### 7.2 Clinical manifestations

Clinical features with the required robustness were rarely described in patients with idiosyncratic drug-induced anti-CYP autoimmune hepatitis unless RUCAM was used (**Table 8**). Limited to RUCAM-based cases due to sevoflurane, comprehensive clinical features were described (15, 125). Manifestations include fever, jaundice, flu-like symptoms, vomiting, right upper quadrant abdominal pain, rash, reduced appetite, and myalgias after the second anesthesia (125); fever was rarely reported (15).

### 7.3 Laboratory data

For RUCAM-based cases of idiosyncratic anti-CYP autoimmune hepatitis caused by sevoflurane, maximum serum ALT activities were 204 U/L (15) and 429 U/L (125). The analysis of all RUCAM-based cases due to sevoflurane (15, 125, 131) revealed that, in some patients, serum titers of anti-CYP2E1 antibodies were not detectable (15, 131).

### 7.4 Liver histology

Liver biopsy was rarely performed to obtain hepatic histology, which is known for its lack of specificity in iDILI cases and, therefore, it is not a RUCAM element and is not required for RUCAM assessment (5, 7). In rare sevoflurane cases with acute or prolonged clinical courses, liver histology showed, in one patient, acute hepatitis with centrilobular necrosis, hemorrhage, rosetting of liver cells, minimal interface hepatitis, and bridging necrosis, while in a second patient, eight months after anesthesia, resolving liver injury was reported; in a third patient, eight months after the last anesthesia, signs of mild lobular and periportal inflammation with mild fibrosis prevailed (125).

### 7.5 Treatment and prognosis

RUCAM-based idiosyncratic drug-induced anti-CYP autoimmune hepatitis commonly resolves after the acute phase with a good prognosis, but few patients experienced prolonged clinical courses of chronic hepatitis with transition to cirrhosis, primarily resulting from repeated sevoflurane anesthesias or occupational exposures (15, 125). Patients with persistently elevated serum ALT activities are commonly treated with immunosuppressant agents such as prednisolone, azathioprine, and rituximab (125).

### 7.6 Molecular pathomechanisms

Sevoflurane is predominantly metabolized by the hepatic microsomal CYP2E1 isoform and undergoes biotransformation to organic and inorganic fluoride metabolites (132, 133). Due to its specific chemical structure and unique low hepatic metabolic rate, it was proposed that sevoflurane does not result in the formation of trifluoroacetylated liver proteins and therefore cannot stimulate the formation of anti-trifluoroacetylated protein antibodies, in line with published work on the absence of sevoflurane modification of liver proteins by covalent binding; however, this condition may be attributed to methodological problems resulting from the failure to detect low intermediate amounts (132). These theoretical considerations on trifluoroacetyl (TFA) are seemingly now outdated in view of the clinical observation that sevoflurane causes not only liver injury but also serum anti-TFA antibodies and anti-CYP2E1 antibodies (**Table 8**) (15, 125). These antibodies were seen in only some patients at admission, and it may take more time for the autoantibodies to be generated at detectable levels (15). There is now sufficient evidence that sevoflurane can produce antibodies to both trifluoroacetylated phospholipid and protein adducts and to CYP2E1, a basic requirement for idiosyncratic drug-induced anti-CYP autoimmune hepatitis due to sevoflurane (15, 125).

## 8 HLA-based immune iDILI

### 8.1 Basic aspects

A minor part of iDILI cases is due to genetic factors related to human leukocyte antigen (HLA) allele variability and is classified as a special type of iDILI termed HLA-based immune iDILI (134), which should be differentiated from other immune and autoimmune iDILI types (**Table 1**).

### 8.2 Diagnosis

HLA genetics were verified for 19 drugs and one drug class in a total of 900 cases of HLA-based immune iDILI with causal evidence based on RUCAM, as reported in 16 publications (**Table 9**) (135–151).

**Table 9:** Drugs implicated in HLA-based immune iDILI with RUCAM-based causality.

DRUG	HLA allele	RUCAM-based iDILI cases (*n*)	RUCAM-based causality grading	References
Amoxicillin	A*01:01 C*03:02 B*58:01 DPB1*01:01	15	Not specified	Nicoletti, 2019 (135)
Amoxicillin- Clavulanate	A*02:01 DQB1*06:02	201	14/201 patients had a possible causality, and 187 a probable or highly probable causality grading	Lucena, 2011 (136)
Amoxicillin- Clavulanate	A*30:02 B*18:01 DRB1*15:01 DQB1*06:02	75	Possible causality and higher gradings	Stephens, 2013 (137)
Amoxicillin- Clavulanate	DRB1*15:01	14	Not specified	O’Donohue, 2000 (138)
Antituberculotics + Antiretrovirals	B*57:02 B*57:03	46	4/46 patients had a possible causality grading, 12 a probable, and 30 a highly probable causality	Petros, 2017 (139)
Carbamazepine	A*31:01	29	All patients had a possible causality and higher	Nicoletti, 2019 (140)
Dapsone	B*13:01	4	Highly probable causality	Devarbhavi, 2022 (141)
Enalapril	A*33:01	4	Not specified	Nicoletti, 2017 (142)
Erythromycin	A*33:01	10	Not specified	Nicoletti, 2017 (142)
Fenofibrate	A*33:01	7	Not specified	Nicoletti, 2017 (142)
Flucloxacillin	B*5701	51	4/51 patients had a possible causality, 18 a probable causality, and 29 a highly probable causality grading	Daly, 2009 (143)
Flucloxacillin	B*57:01	6	2/6 patients had a possible causality, 2 a probable, and 2 a highly probable causality	Monshi, 2013 (144)
Flucloxacillin	B*57:01	197	22/197 patients had a possible causality, 90 a probable causality, and 85 a highly probable causality grading	Nicoletti, 2019 (135)
Flucloxacillin	B*57:01	1	Score 8, probable causality	Teixera, 2020 (145)
Flupirtine	DRB1*16:01DQ B*05:02	11	1/11 patients had an unlikely causality grading, 5 a possible, and 5 a probable causality grading	Nicoletti, 2016 (146)
Infliximab	B*39:01	18	Not specified	Bruno, 2020 (147)
Isoxazolyl penicillins	C*07:04 DQB1*06:09	6	Not specified	Nicoletti, 2019 (135)
Methimazole	C*03:02	40	1/40 patients had a possible causality grading, 37 a probable, and 2 a highly probable causality grading	Li, 2019 (148)
Methyldopa	A*33:01	4	Not specified	Nicoletti, 2017 (142)
Minocycline	B*35:02	25	Not specified	Urban, 2017 (149)
Nitrofurantoin	A*33:01 DQB1*02:02 A*30:02 DQA1*02:01 DRB1*07:01 DPB1*16:01 C*06:02	26	18/26 patients had a score of above 6, in line with a probable or highly probable causality	Daly, 2023 (150)
Sertaline	A*33:01	5	Not specified	Nicoletti, 2017 (142)
Terbinafine	A*33:01	14	Not specified	Nicoletti, 2017 (142)
Ticlopidine	A*33:01	5	Not specified	Nicoletti, 2017 (142)
Trimethoprim-sulfamethoxazole	B*14:01 B*14:02 B*35:01	86	Not specified	Li, 2021 (151)

The table is retrieved from an earlier report published in an open-access journal (134). Abbreviations: iDILI, idiosyncratic drug-induced liver injury; HLA, human leukocyte antigen; RUCAM, Roussel–Uclaf Causality Assessment Method.

In 683/900 iDILI cases (76%), RUCAM-based final scores or causality gradings were presented, ranging from possible to highly probable levels of causality in most study cohorts (**Table 9**). The inclusion of cases with a possible causality ranking remains problematic, as this confounds valid cohort results obtained from cases with a probable or highly probable causality level. Possible causality levels commonly are due to a retrospective study protocol with incomplete data collection and the neglect of alternative causes, thus calling for prospective studies as the best analytical approach (7). Among the drugs most implicated in RUCAM-based iDILI with HLA analysis, amoxicillin–clavulanate was the most frequently identified, followed by flucloxacillin, trimethoprim–sulfamethoxazole, methimazole, carbamazepine, and nitrofurantoin, with case numbers ranging from 1 to 201 (**Table 9**).

Drugs causing iDILI cases with unverified diagnoses and suspected HLA associations. Highly problematic were studies on HLA alleles in cases of iDILI that were not assessed for causality using RUCAM but were instead assessed using the nonvalidated Drug-Induced Liver Injury Network (DILIN) method based on arbitrary subjective opinion (**Table 10**) (93, 152–162).

**Table 10:** Drugs causing iDILI evaluated for underlying HLA association but not assessed by RUCAM.

DRUG	HLA allele	iDILI cases (*n*)	Causality assessment method	References
Allopurinol	A*34:02 B*53:01 B*58:01	11	No RUCAM but DILIN method	Fontana, 2021 (152)
Allopurinol	B*58:01	3	None	Kim, 2017 (153)
Amoxicillin- Clavulanate	DRB1*1501 DQB1*0602	35	None	Hautekeete, 1999 (93) Meng, 2016 (154)
Halothane	*DR2*	14	None	Otsuka, 1985 (155)
Lapatinib	DRB1*07:01	65	None	Tangamornsuksan, 2020 (156)
Lumiracoxib	DRB1*15:01	139	None	Singer, 2010 (157)
Nitrofurantoin	DRB1*11:04	78	No RUCAM but DILIN method	Chalasani, 2023 (158)
Pazopanib	B*57:01 C*04:01 C*06:02	2,190	None	Xu, 2016 (159)
Terbinafine	A*33:01	15	No RUCAM but DILIN method	Fontana, 2018 (160)
Ticlopidine	A*33:03	22	None	Hirata, 2008 (161)
Ximelagatran	DRB1*07 DQA1*02	74	None	Kindmark, 2008 (162)

Some cases were characterized by severe cutaneous adverse reactions (SCARs), such as Stevens–Johnson syndrome (SJS), toxic epidermal necrolysis (TEN), and drug reaction with eosinophilia and systemic symptoms (DRESS) (134). Abbreviations: DILIN, Drug-Induced Liver Injury Network; iDILI, idiosyncratic drug induced liver injury; HLA; human leukocyte antigen; RUCAM, Roussel Uclaf Causality Assessment Method.

It is obvious that data derived from publications of suspected HLA-based immune iDILI lacking a robust causality assessment cannot be used for scientific and clinical discussions (**Table 10**). Indeed, such reports are a waste of energy and financial resources provided by governmental agencies, institutes, and hospitals.

Although cases of iDILI due to many drugs showed an association and possibly a causal relationship with HLAs (**Table 9**) (135–150, 163), no significant HLA association was detectable for some drugs and drug classes implicated in causing iDILI, as discussed and referenced previously (134) (**Table 11**).

**Table 11:** Drugs causing iDILI with lack of detectable HLA association.

Drugs with iDILI and no detectable significant signal in HLA region
Atorvastatin and other statins Fasiglifam (TAK-875) Azathioprine and other thiopurines Interferon beta Ciprofloxacin and other fluoroquinolones Isoniazid Diclofenac Nimesulide

### 8.3 Clinical manifestations

Clinical features of HLA-based immune iDILI were rarely described in large cohorts focused on HLA details and characterized by the heterogeneity of the various drugs and drug classes included in the study cohorts but are best described using data from single case reports assessed by RUCAM with reference to a single drug. As an example, asthenia, anorexia, nausea, abdominal discomfort, fever, jaundice, pruritus, and choluria were reported in a patient with HLA-based immune iDILI due to flucloxacillin treatment, with HLA-B* 5701 allele association and a RUCAM score of 8, in line with a probable causality grading (145). In the cohort consisting of patients with HLA-based immune iDILI due to amoxicillin–clavulanate, jaundice was reported in 21/75 cases (28%) (137).

### 8.4 Laboratory data

In the flucloxacillin patient, the serum activity of ALT was 646 U/L, and total bilirubin was 3.3 mg/dL, ascertained by RUCAM (145). The HLA-based immunome iDILI cohort comprising amoxicillin–clavulanate cases reported ALT values of 19.5× upper limit of normal (ULN) and ALP values of 2.3× ULN, while total bilirubin value of 10.4 mg/dL was presented as the mean value (137). In this study HLA alleles varied and included A*30:02, B*18:01, DRB1*15:01, and DQB1*06:02.

### 8.5 Liver histology

Upon liver biopsy, liver histology in the flucloxacillin patient showed, apart from slight multifocal accentuated changes in the centrilobular areas, sinusoidal dilatation, marked congestion, hemorrhage, and multifocal collapse of hepatocytes, as well as, in the portal areas, not only bridges but also proliferated bile ducts and inflammatory infiltrates of variable density, predominantly of the mononuclear type (145).

### 8.6 Treatment and prognosis

The analysis of HLA-based auto immune iDILI cases with a verified diagnosis by RUCAM revealed little robust data on treatment modalities and prognosis (**Table 9**) (135–151). In suspected cases, cessation of the assumed responsible drug is recommended as an initial approach, in line with other iDILI types. In the amoxicillin–clavulanate study, the clinical outcome was described as severe damage, including acute liver failure and liver transplantation in 2/75 cases (2.7%) (137).

### 8.7 Molecular pathomechanisms

Mechanistic and molecular sequelae of HLA-based immune iDILI due to flucloxacillin were thoroughly analyzed, as this drug is among the top-ranking causes of iDILI (144, 163). With respect to hepatocellular iDILI due to flucloxacillin, with evidence based on RUCAM, there is extensive cross-talk among the HLA B*57:01, the metabolic CYP 3A4/3A7 pathway involved in flucloxacillin degradation, and immune mechanisms leading to the HLA-based immune iDILIs (163). Studies were expanded to investigate the HLA-B*57:01-restricted activation of drug-specific T cells, which provides the immunological basis for flucloxacillin-induced liver injury (144). For flucloxacillin, a delay in reaction onset and the identification of HLA-B*57:01 as a susceptibility factor are suggestive of an immune pathogenesis. Characterization of flucloxacillin-responsive CD41+ and CD81+ T cells from patients with liver injury revealed that naïve CD45RA1+CD81+ T cells from volunteers expressing HLA-B*57:01 are activated by flucloxacillin when dendritic cells present the drug antigen. T-cell clones expressing CCR4 and CCR9 migrated toward CCL17 and CCL 25 and secreted interferon-gamma (IFN-γ), T helper (Th) 2 cytokines, perforin, granzyme B, and FasL following drug stimulation. Flucloxacillin bound covalently to selective lysine residues on albumin in a time-dependent manner, and the level of binding correlated directly with the stimulation of clones. Activation of CD8+ clones with flucloxacillin was processing-dependent and restricted by HLA-B*57:01 and the closely related HLA-B*58:01. Clones displayed additional reactivity against β-lactam antibiotics, including oxacillin, cloxacillin, and dicloxacillin, but not abacavir or nitroso sulfamethoxazole (144). This study provides the immune basis for flucloxacillin-induced liver injury and links the genetic association to the disease.

## 9 Immune iDILI with SJS/TEN

### 9.1 Basic aspects

Drugs can trigger the immune iDILI with Stevens–Johnson syndrome (SJS) and toxic epidermal necrolysis (TEN) as a special type of iDILI (**Table 1**), representing immune-based variant disorders within a continuous spectrum, with milder forms classified as SJS and SJS/TEN overlap and TEN representing the most serious form (164). For reasons of simplicity, the term SJS/TEN is now commonly now and includes SJS, SJS/TEN overlap, and TEN. SJS is defined as a skin reaction involving less than 10% of the body surface area (BSA), whereas TEN is characterized by skin involvement of more than 30% of the BSA, while the intermediate form is classified as involving 10%–30% of the BSA (165–168). With the exception of BSA extent and severity grade, many features are similar among SJS, SJS/TEN overlap, and TEN; therefore, the three entities are now collectively referred to as SJS/TEN (165, 169, 170). Agreement exists that the previously termed intermediate form should now be designated as SJS/TEN overlap (166, 171–173).

### 9.2 Diagnosis

Immune iDILI with SJS and TEN is composed of two major disease entities, iDILI and, collectively, the SJS/TEN, requiring two different diagnostic algorithms to ascertain causality for the implicated drug (164). The iDILI component is now best assessed using the updated RUCAM (7), whereas the ALDEN diagnostic algorithm, published in 2010, is commonly used for the SJS/TEN component (12). Retrieved from cases with diagnoses ascertained by RUCAM and, in virtually all cases, additionally verified using the ALDEN diagnostic method, a list of drugs implicated in immune-based iDILI with SJS/TEN is provided (**Table 12**) (174–179).

**Table 12:** Selected drugs implicated in immune iDILI with SJS/TEN.

Drugs/drug classes	Cases (*n*)	Causality algorithm	Outcome	References
Allopurinol	2	RUCAM + ALDEN +	All 2 survived	Devarbhavi, 2016 (174)
Allopurinol	1	RUCAM + ALDEN +	N.A.	Zhang, 2020 (175)
Amoxicillin	N.A.	RUCAM + ALDEN -	Cases of acute liver failure	Ortega-Alonso, 2017 (176)
Ampicillin	N.A.	RUCAM + ALDEN -	Cases of acute liver failure	Ortega-Alonso, 2017 (176)
Aspirin	1	RUCAM + ALDEN +	N.A.	Zhang, 2020 (175)
Carbamazepine	2	RUCAM + ALDEN +	2/2 died	Devarbhavi, 2016 (174)
Carbamazepine	8	RUCAM + ALDEN +	N.A.	Zhang, 2020 (175)
Carbamazepine	36	RUCAM + ALDEN +	4/36 died	Devarbhavi, 2023 (177)
Ceftazidime	1	RUCAM + ALDEN +	N.A.	Zhang, 2020 (175)
Ceftriaxone	1	RUCAM + ALDEN +	Lethal outcome	Devarbhavi, 2016 (174)
Ceftriaxone	1	RUCAM + ALDEN +	N.A.	Zhang, 2020 (175)
Celecoxib	N.A.	RUCAM + ALDEN -	No cases of acute liver failure	Ortega-Alonso, 2017 (176)
Clobazam	2	RUCAM + ALDEN +	1/3 died	Devarbhavi, 2023 (176)
Clonazepam	2	RUCAM + ALDEN +	All survived	Devarbhavi, 2023 (176)
Cotrimoxazole	3	RUCAM + ALDEN +	All 3 survived	Devarbhavi, 2016 (174)
Celecoxib	N.A.	RUCAM + ALDEN -	No cases of acute liver failure	Ortega-Alonso, 2017 (176)
Dapsone	5	RUCAM + ALDEN +	3/5 died	Devarbhavi, 2016 (174)
Fluoxetine	1	RUCAM + ALDEN +	Survived	Agrawal, 2019 (178)
Gabapentin	1	RUCAM + ALDEN +	Survived	Devarbhavi, 2023 (177)
Ibuprofen	N.A.	RUCAM + ALDEN -	Cases of acute liver failure	Ortega-Alonso, 2017 (176)
Lamotrigine	1	RUCAM + ALDEN +	Lethal outcome	Devarbhavi, 2016 (174)
Lamotrigine	1	RUCAM + ALDEN +	N.A.	Zhang, 2020 (175)
Lamotrigine	3	RUCAM + ALDEN +	1/3 died	Devarbhavi, 2023 (177)
Leflunomide	3	RUCAM + ALDEN +	All 3 died	Devarbhavi, 2016 (174)
Leflunomide	2	RUCAM + ALDEN +	N.A.	Zhang, 2020 (175)
Levitericetam	1	RUCAM + ALDEN +	Lethal outcome	Devarbhavi, 2016 (174)
Levitericetam	3	RUCAM + ALDEN +	All survived	Devarbhavi, 2023 (177)
Levofloxacin	1	RUCAM + ALDEN +	Survived	Devarbhavi, 2016 (174)
Nevirapine	6	RUCAM + ALDEN +	All survived	Devarbhavi, 2016 (174)
Omeprazole	1	RUCAM + ALDEN +	N.A.	Zhang, 2020 (175)
Oxacarbazepine	1	RUCAM + ALDEN +	Survived	Devarbhavi, 2016 (174)
Oxacarbazepine	2	RUCAM + ALDEN +	N.A.	Zhang, 2020 (175)
Paracetamol	1	RUCAM + ALDEN +	N.A.	Zhang, 2020 (175)
Penicillin	1	RUCAM + ALDEN +	N.A.	Zhang, 2020 (175)
Phenobarbitone	2	RUCAM + ALDEN +	1/2 died	Devarbhavi, 2016 (174)
Phenobarbitone	1	RUCAM + ALDEN +	N.A.	Zhang, 2020 (175)
Phenobarbitone	8	RUCAM + ALDEN +	2/8 died	Devarbhavi, 2023 (177)
Phenylbutazone	2	RUCAM + ALDEN +	N.A.	Zhang, 2020 (175)
Phenytoin	2	RUCAM + ALDEN +	1/2 died	Devarbhavi, 2016 (174)
Phenytoin	1	RUCAM + ALDEN +	N.A.	Zhang, 2020 (175)
Phenytoin	71	RUCAM + ALDEN +	4/71 died	Devarbhavi, 2023 (177)
Tegafur	1	RUCAM + ALDEN +	N.A.	Zhang, 2020 (175)
Terbinafine	N.A.	RUCAM + ALDEN -	Cases of acute liver failure	Ortega-Alonso, 2017 (176)
Topiramate	1	RUCAM + ALDEN +	Survived	Devarbhavi, 2023 (177)
Valproate	14	RUCAM + ALDEN +	1/14 died	Devarbhavi, 2023 (177)
Warfarin	1	RUCAM + ALDEN +	Survived	Xiong, 2021 (179)
Zonisamide	1	RUCAM + ALDEN +	Survived	Devarbhavi, 2023 (177)

Compilation of selected drugs implicated in causing immune iDILI with SJS and TEN. For all listed drugs, causality of DILI for the culprit drug was verified using the RUCAM scoring algorithm, and for most drugs, the diagnosis of SJS/TEN was verified using the ALDEN scoring algorithm. The listing was confined to conventional drugs, excluding herbal medicines such as Traditional Chinese Medicines (TCM), because these products cause herb-induced liver injury (HILI) rather than DILI. The + sign indicated that the specific diagnostic algorithm was used to verify the diagnosis, whereas the − sign signified that the specific algorithm was not applied. Abbreviations: ALDEN, Algorithm for Drug Causality for Epidermal Necrolysis; N.A., not available; RUCAM, Roussel Uclaf Causality Assessment Method.

The analysis of published case data revealed convincingly that, for the clinically important cohort of immune-based iDILI with SJS/TEN, RUCAM and ALDEN were used together in 5/6 reports (83.3%) and provided good results for the diagnosis (**Table 12**) (174–179). This approach provided firm evidence in 203 cases that the suspected drugs were indeed the culprit medications. The listing also shows which drugs carry a high risk of causing death in patients (**Table 12**). The single study that applied only RUCAM ignored the value of ALDEN and thereby provided results that were not evidence-based (**Table 12**) (176). Thus, for reasons of completeness and strong evidence, in SJS/TEN patients with suspected iDILI, the use of both algorithms—the updated RUCAM and the ALDEN method—should be obligatory in future studies and regarded as the primary gold standard. There were also reports of drugs viewed as causative or noncausative for SJS/TEN and assessed only by the ALDEN tool (164); however, these cases differ from the cohort of immune-based iDILI with SJS/TEN because they were not assessed using RUCAM (**Table 13**) (12, 180).

**Table 13:** Drugs or drug classes implicated or not implicated in SJS or TEN as verified by ALDEN.

Drugs or drug classes	Cases (*n*)	Causality method	References
ACE Inhibitors	0	ALDEN +	Sassolas, 2020 (12)
Acetylsalicylic acid	0	ALDEN +	Sassolas, 2010 (12)
Acetaminophen	8	ALDEN +	Sassolas, 2010 (12)
Allopurinol	5	ALDEN +	Sassolas, 2010 (12)
Allopurinol	11	ALDEN +	Gronich, 2022 (180)
Amoxicillin	6	ALDEN +	Gronich, 2022 (180)
Amoxicillin-clavulanate	4	ALDEN +	Gronich, 2022 (180)
Acyclovir	1	ALDEN +	Gronich, 2022 (180)
Bendamustine	2	ALDEN +	Gronich, 2022 (180)
Benzodiazepines	0	ALDEN +	Sassolas, 2010 (12)
Beta-Blockers	0	ALDEN +	Sassolas, 2010 (12)
Calcium channel blockers	0	ALDEN +	Sassolas, 2010 (12)
Cabozantinib	1	ALDEN +	Gronich, 2022 (180)
Carbamazepine	2	ALDEN +	Gronich, 2022 (180)
Carfilzomib	1	ALDEN +	Gronich, 2022 (180)
Cefazolin	1	ALDEN +	Gronich, 2022 (180)
Ceftriaxone	1	ALDEN +	Gronich, 2022 (180)
Cefuroxime	4	ALDEN +	Gronich, 2022 (180)
Celecoxib	1	ALDEN +	Gronich, 2022 (180)
Ciprofloxacin	6	ALDEN +	Gronich, 2022 (180)
Citalopram	1	ALDEN +	Sassolas, 2010 (12)
Citalopram	1	ALDEN +	Gronich, 2022 (180)
Clindamycin	4	ALDEN +	Gronich, 2022 (180)
Codeine	1	ALDEN +	Gronich, 2022 (180)
Corticosteroids	7	ALDEN +	Sassolas, 2010 (12)
Dipyrone	3	ALDEN +	Gronich, 2022 (180)
Etodolac	3	ALDEN +	Gronich, 2022 (180)
Etoricoxib	5	ALDEN +	Gronich, 2022 (180)
Fluconazole	2	ALDEN +	Sassolas, 2010 (12)
Fluoxetine	2	ALDEN +	Sassolas, 2010 (12)
H1 anti-histamine	0	ALDEN +	Sassolas, 2010 (12)
HMG-CoA reductases, statins	0	ALDEN +	Sassolas, 2010 (12)
Hydrochloroquine	1	ALDEN +	Gronich, 2022 (180)
Ibuprofen	0	ALDEN +	Sassolas, 2010 (12)
Ibuprofen	1	ALDEN +	Gronich, 2022 (180)
Ketoprofen	3	ALDEN +	Sassolas, 2010 (12)
Leflunomide	1	ALDEN +	Sassolas, 2010 (12)
Lamotrigine	1	ALDEN +	Sassolas, 2010 (12)
Lamotrigine	9	ALDEN +	Gronich, 2022 (180)
Levomepromazine	1	ALDEN +	Gronich, 2022 (180)
Macrogol	1	ALDEN +	Gronich, 2022 (180)
Metamizole	2	ALDEN +	Sassolas, 2010 (12)
Metronidazole	1	ALDEN +	Sassolas, 2010 (12)
Naproxen	1	ALDEN +	Sassolas, 2010 (12)
Nimesulide	1	ALDEN +	Sassolas, 2010 (12)
Nitrates	0	ALDEN +	Sassolas, 2020 (12)
Nitrofurantoin	1	ALDEN +	Gronich, 2022 (180)
Ofloxacin	1	ALDEN +	Gronich, 2022 (180)
Paroxetine	1	ALDEN +	Sassolas, 2010 (12)
Phenylbutazone	1	ALDEN +	Sassolas, 2010 (12)
Phenylbutazone and Kebuzone	3	ALDEN +	Sassolas, 2010 (12)
Phenytoin	1	ALDEN +	Sassolas, 2010 (12)
Phenytoin	8	ALDEN +	Gronich, 2022 (180)
Pralatrexate	1	ALDEN +	Gronich, 2022 (180)
Pregabalin	1	ALDEN +	Gronich, 2022 (180)
Pyrazolone analgesics	6	ALDEN +	Sassolas, 2010 (12)
Quetiapine	1	ALDEN +	Gronich, 2022 (180)
Roxithromycin	3	ALDEN +	Gronich, 2022 (180)
Spironolactone	0	ALDEN +	Sassolas, 2010 (12)
Sulfamethoxazole	1	ALDEN +	Sassolas, 2010 (12)
Sulfasalazine	1	ALDEN +	Gronich, 2022 (180)
Sulfonylurea antidiabetics	0	ALDEN +	Sassolas, 2010 (12)
Sunitinib	1	ALDEN +	Gronich, 2022 (180)
Terbinafine	1	ALDEN +	Gronich, 2022 (180)
Thiabendazole	2	ALDEN +	Sassolas, 2010 (12)
Thiazide diuretics	0	ALDEN +	Sassolas, 2010 (12)
Thioacetazone	1	ALDEN +	Sassolas, 2010 (12)
Topiramate	1	ALDEN +	Gronich, 2022 (180)
Tramadol	0	ALDEN +	Sassolas, 2010 (12)
Trimethoprim sulfamethoxazole	4	ALDEN +	Gronich, 2022 (180)
Valproic acid	4	ALDEN +	Gronich, 2022 (180)
Valproic acid	3	ALDEN +	Sassolas, 2010 (12)
Vancomycin	3	ALDEN +	Gronich, 2022 (180)
Vasodilators	0	ALDEN +	Sassolas, 2010 (12)

List of drugs, most of which were implicated in SJS/TEN (12, 180) as assessed by the ALDEN tool (12). The + sign indicates that the ALDEN tool was used to verify the diagnosis of SJS/TEN. Table taken from a previous report published in an open-access journal (164). Abbreviations: ALDEN, Algorithm for Drug Causality for Epidermal Necrolysis.

Theoretically, cases assessed only by the ALDEN tool only may have an iDILI component (**Table 13**) (12, 180), that was not recognized because RUCAM (5–7) was not applied. Additional assessment using RUCAM might have substantially increased the number of drugs currently identified as causing immune-based iDILI with SJS and TEN, as listed above (**Table 12**). There were other cohorts of drugs causing suspected but unverified immune-based iDILI with SJS and TEN in which causality assessment was either lacking or based on problematic assessment tools that had not been validated by positive exposure results, as done for RUCAM in 1993 (5, 6) (164).

### 9.3 Clinical manifestations

Clinical features of immune-based iDILI with SJS/TEN were described in most reports providing cases with a firm diagnosis (**Table 1**) (174–179). Clinical manifestations included jaundice, ascites, and encephalopathy as signs of severe liver injury, in addition to dermal features associated with SJS/TEN (174), as well as fatigue, inappetence, yellow discoloration of the urine, pruritis, fever, and yellow staining of the skin and sclera (175). The interval between drug exposure and the onset of the skin reaction was variable and could be as long as 50 days, important clinical information that may help avoid missing the diagnosis (174). A major diagnostic issue remains for the immune-based iDILI with SJS/TEN and for all cases of SJS/TEN because many non-drug compounds may confound the diagnosis (**Table 14**) (164, 181–189).

**Table 14:** Non-drug culprits implicated in causing SJS/TEN.

Non-drug culprit	Cases(*n*)	Comments	References
Acetochlor	1	Industrial chemical	Yang, 2018 (181)
Arsenic	1	Heavy metal	Yang, 2018 (181)
Biological	2	Vaccine	Wang, 2022 (182)
Carbamate	1	Occupational exposure to this insecticide	Lim, 2010 (183)
Cardiac catheterization dye	1	Not further specified	Wang, 2022 (182)
Chemical substance	10	Arsenic (2x) Dimethyl cyanocarbonimidodithionate (1x)Carbamate insecticide (2x) Gangliosides (1x) Iodine (1x) Mercury (1x) Organophosphate insecticide (1x)Trichloroethylene (1x)	Wang, 2022 (182)
Chinese patent medicines	18	Not specified	Wang, 2022 (182)
Contrast medium as diagnostic	9	Not further specified	Wang, 2022 (182)
Coxsackie virus A6	8	Identified as CVA 6 in blistering skin lesions (6x) and isolated by a throat swab (2x)	Chung, 2013 (184)
Diatrizoate meglumine diatrizoate sodium	1	Known as Gastrografin, used for oral radiographic examination of esophagus, stomach, proximal small intestine, and colon	Wang, 2022 (182)
Enterovirus	1	Acquired in a stable	De Guido, 2020 (185)
Glyphosate	1	Following inhalation of this herbicide, short treatment with aspirin, paracetamol, and chlorpheniramine	Voltan, 2010 (186)
Hair dry	1	Not specified	Kim, 2012 (187)
Hepatitis A	1	Hepatitis A virus (HAV) was assumed by error as cause of cirrhosis. However, HAV never causes chronic liver disease like incipient cirrhosis	Zang, 2023 (188)
Herbal medicines	445	Not further specified	Wang, 2022 (182) Kim, 2012 (188)
Herbal medicines	7	Ayurvedic medicines (3x) Golden health blood purifyingtablets [1x)Moringa oleifera (1x) Ophiopogonis tuber (1x) Traditional Chinese Medicines (TCM) (1x)	Wang, 2022 (182)
Infections	25	Brucella melitensis (1x)Cytomegalovirus infection (1x) Dengue virus (1x) Enterovirus (1x) Epstein-Barr virus infection (1x) Herpes simplex virus (4x) Influenza B infection (2x) Mucor infection (1x) Parvovirus infection (1x) Pneumonia infection (1x) Psittacosis (1x) Respiratory infection (2x)Staphylococcus septicemia (1x) Upper respiratory infection (1x)Varicella infection (1x) Varicella-zoster virus (1x) Viral hepatitis A (1x) Viral illness (2x) Yersinia enterocolica infection (1x)	Wang, 2022 (182)
Mycoplasma pneumoniainfection	44	Highest frequency in patients with Stevens-Johnson Syndrome	Wang, 2022 (182)
Naphthalenedisulfonic acid dimethyl ester	1	Industrial chemical	Yang, 2018 (181)
Others	25	Various diseases and other causes specified	Wang, 2022 (182)
Others	39	Not specified	Kim, 2012 (187)
Radiotherapy	29	Brain radiotherapy (15x) Unspecified radiotherapy (1x) and associated with drug use in 20 patients	Wang, 2022 (182)
Trichloroethylene	1	Industrial chemical	Yang, 2018 (181)
Vaccines	9	Vaccine against: Anthrax (1x) Hanta virus (1x) Measles (1x)MPR (1x) Rabies (1x) Small pox (1x) Tetanus (1x) Varicella zoster virus (1x) Yellow fever (1x)	Wang, 2022 (182)
Vitamins	3	Pyritinol (1x) Supradyn (1x) Vitamin B complex (1x)	Wang, 2022 (182)
Ultraviolet radiation	13	Combined with these drugs:Carbamazepine (1x) Chloroquine (1x) Ciprofloxacin (1x) Hydroxychloroquine (3x) Ibuprofen (1x) Itraconazole (1x) Lamotrigine (2x) Naproxene (1x) Sulfasalazine (1x) Tramadol (1x)	McKinley, 2023 (189)

Table taken from a previous report published in an open-access journal (164). Abbreviations: MPR, measles, parotitis, and rubella; NSAIDs, nonsteroidal anti-inflammatory drugs; SJS/TEN, Stevens–Johnson syndrome/toxic epidermal necrolysis.

Clinical manifestations are often described in cases with unidentified culprits, which be viewed as confounders, because 5%–35% of cases remain idiopathic (12, 168, 190–192). Many tests are warranted to identify the cause of SJS and clarify the etiology of cases currently classified as idiopathic (193). An overview of reports dealing with unidentified causes of SJS/TEN is provided in a listing (**Table 15**) (12, 129, 168, 185, 191, 194–196).

**Table 15:** Unidentified culprits in SJS/TEN.

References	SJS, TEN alone, or together	Cases (*n*)	Diagnostic causality algorithm	Details and comments
Zimmerman, 2019 (168)Wolff, 2012 (190)	SJS/TEN	N.A.	N.A.	Discussed is the fact that 5-20% of cases remain idiopathic
Sassolas, 2010 (12)	SJSTEN	N.A.	ALDEN	In 65% of SJS and TEN, drugs were implicated as opposed to 35% with nondrug unidentified culprits
Diphoorn, 2016 (191)	SJS/TEN	76	ALDEN	No drug a causative was found in 6.6% of cases
Bang, 2012 (192)	SJS	N.A.	SCORTEN Naranjo	More than 80% of SJS were caused by drugs and 20% by non-drug unidentified culprits
De Guido, 2020 (193)	SJS	N.A.	N.A.	Discussed is the role of drugs in 53-95% of cases, of infections in 5-31%, and idiopathic in 5-18%
Nozaki, 2015 (194)	SJS	8	SCORTEN	Therapy study was done in all non-drug cases
Shanbhag, 2020 (195)	SJS/TEN	N.A.	N.A.	Mentioned is the fact that no drug origin could be identified in 15% of cases
Cheung, 2024 (196)	SJS/TEN	124	ALDEN	No cause was identified in 4.8% of cases

Table taken from a previous report published in an open-access journal (164). Abbreviations: ALDEN, Algorithm for Drug Causality for Epidermal Necrolysis; N.A., not available; SCORTEN, Score of Toxic Epidermal Necrolysis; SJS, Stevens–Johnson syndrome; SJS/TEN, Stevens–Johnson syndrome/toxic epidermal necrolysis; TEN, toxic epidermal necrolysis.

SJS/TEN presents with five different cohort types (164): SJS/TEN type 1, which refers to a cohort of SJS/TEN caused by drugs and assessed using both ALDEN and RUCAM (**Table 12**); type 2, representing SJS/TEN due to drugs and assessed only by ALDEN, but not by RUCAM (**Table 13**); type 3, which includes a cohort of SJS/TEN caused by drugs and assessed by non-ALDEN and non-RUCAM tools (**Table 14**); type 4, which focuses on a cohort of SJS/TEN caused by non-drug culprits and assessed using various tools (**Table 15**); and type 5, which considers a cohort of SJS/TEN caused by unknown culprits (164). Using this new SJS/TEN typology will help characterize individual features more precisely, personalize treatment, and clarify pathogenetic mechanisms for each of the five disease types. The new SJS/TEN typology provides clarity by replacing cohort heterogeneity with cohort homogeneity.

### 9.4 Laboratory data

As expected, LTs were abnormal: serum ALT activities were up to 1,207 U/L, AST activities were up to1,454 U/L, and ALP activities were up to 3,120 U/L, with total bilirubin levels reaching up to 17.2 mg/dL (174). Somewhat lower values were reported in another study (175).

### 9.5 Liver histology

Because liver histology is not a diagnostic element of RUCAM (5–7) or the ALDEN algorithm (12), such data are not commonly expected during clinical evaluation (174–179). However, liver histology data were obtained through a postmortem liver biopsy of a patient with immune-based iDILI with SJS/TEN due to lamotrigine, which revealed cholestasis and a scanty infiltrate, and from another patient treated with carbamazepine, whose postmortem examination showed severe portal inflammation with lymphocytes, polymorphonuclear, plasma cells, and occasional eosinophils (174).

### 9.6 Treatment and prognosis

Therapy was initiated by immediate cessation of all suspected drugs, followed by intravenous methylprednisolone at a daily dose of 40 mg/d–100 mg/d in the early stage, with gradual tapering to oral corticosteroids until drug withdrawal, and some patients received intravenous immunoglobulin (IVIG) at a dose of 400 mg/kg/day for five days (175). Overall lethality was 36%, which increased to 45.5% in the presence of jaundice but was lower in children (11%) and patients infected with the human immunodeficiency virus (12.5%) (174). In another study, the lethality rate was 10% (175). Outcome differed from drug to drug, ranging from survival to lethality (**Table 12**), and also depended on the severity of the disease spectrum, as assessed by the Score of Toxic Epidermal Necrolysis (SCORTEN) (171). However, the optimum therapy remains controversial (197–199).

### 9.7 Molecular pathomechanisms

SJS/TEN is a complex disease spectrum (197–199) characterized by disruption that primarily targets the skin and secondarily affects non-skin organs, including the liver (50%), respiratory system, including the lungs (40%), and kidneys (21%) (199). Based on the clinical observation that, in one-third of SJS/TEN patients, malaise, fever, sore throat, and cough precede the onset of the typical skin manifestations by a few days (197–199), there is compelling evidence that the skin is the primary organ triggering this complex disease spectrum. However, a knowledge gap exists regarding the relationship between drug-induced skin alterations and liver injury in the context of overall immune-based iDILI with SJS/TEN (164). On theoretical grounds, it seems that the liver injury observed in a subset of SJS/TEM patients is causally related to several cytokines and cytotoxic proteins that have been shown to be elevated in the blood blister fluid and skin tissue from patients with SJS/TEN, and that a cytokine storm may trigger liver injury (200–220). Open questions relate to the observation that iDILI developed in only half of the SJS/TEN patients (199). The absences of iDILI features may be genuine or may have been overlooked because they developed after a longer interval following the initial skin disruption. Another interesting finding was the occurrence of SJS/TEN triggered by ultraviolet radiation from sun exposure or tanning bed use in patients receiving drug therapy in the absence of concomitant iDILI (189, 201–212). UV radiation may lead to epidermal ROS overproduction, local cytotoxicity, and activation of the immune system, resulting in T-cell recruitment and further ROS production (189).

**Figure 2:**
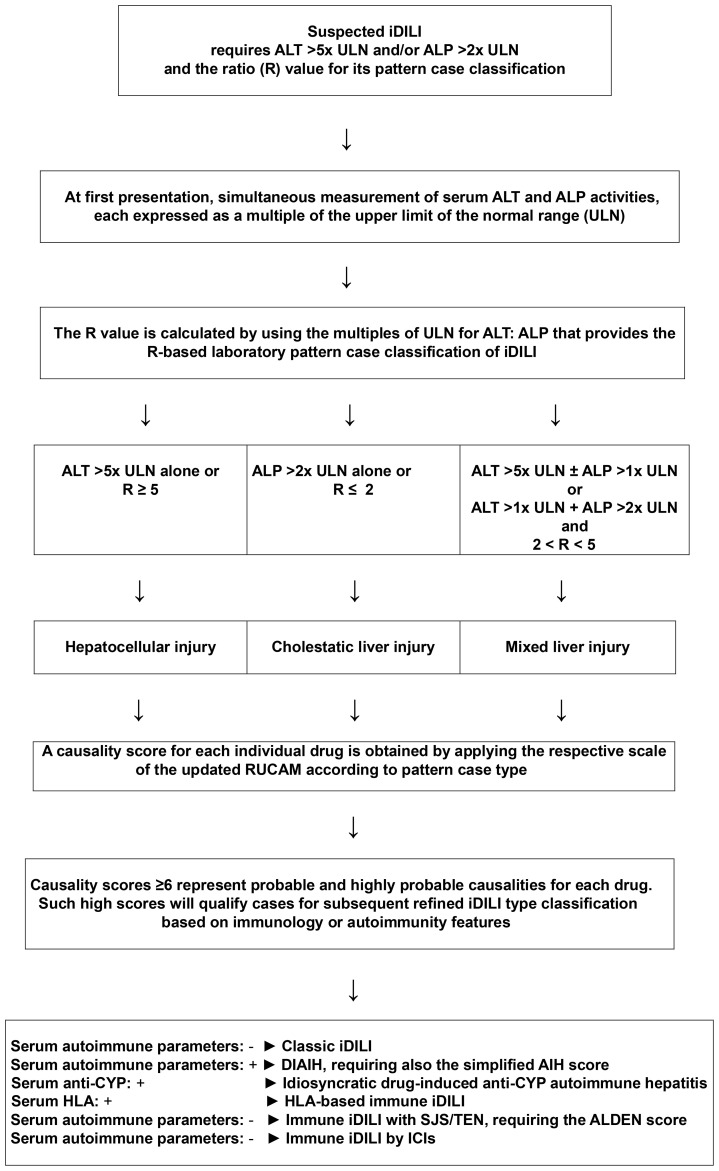
Diagnostic workflow for RUCAM-based refined classification of the immunology and autoimmunology iDILI types. Part of the table was modified from the updated RUCAM report published in an open-access journal (7). The + sign indicated that the specific diagnostic parameter was positive and was used to verify the diagnosis, as opposed to the – sign, which signified that the specific parameter was negative. Abbreviations: AIH, autoimmune hepatitis; ALDEN, Algorithm of Drug Causality for Epidermal Necrolysis; ALP, alkaline phosphatase; ALT, alanine aminotransferase; CYP, cytochrome P450; HLA, human leukocyte antigen; ICIs, immune checkpoint inhibitors; iDILI, idiosyncratic drug-induced liver injury; SJS, Stevens–Johnson syndrome; TEN, toxic epidermal necrolysis; ULN, upper limit of normal; R, ratio; RUCAM, Roussel Uclaf Causality Assessment Method.

Starting with events occurring in the skin of patients experiencing SJS/TEN, several models of immunopathogenesis involving T-cell activation have been proposed (197–199, 213–220), with a focus on three models (197) (1): In the hapten/pro-hapten model, drugs or metabolites generated through non-CYP or CYP pathways form complexes with carrier proteins and are presented as haptenated peptides in the peptide-binding groove of HLA molecules (197). In the epidermis, mRNA expression levels of CYP1A2, CYP3A4, and CYP3A5 were detected in Japanese individuals (220), and epidermal CYP isoforms may generate toxic metabolites via the catalytic circle known from the drug metabolism in the liver (**Figure 2**) (3, 10) (2); According to the p-i concept, drugs bind directly and noncovalently to HLA molecules and T-cell receptors (TCRs) (197) (3); The altered peptide model focuses on drugs that bind to the peptide-binding groove of HLA, resulting in the alteration of the HLA-binding peptide repertoire (197).

In more detail, most drugs and their metabolic intermediates are pro-haptens rather than haptens (197). They acquire immunogenicity by covalently binding to carrier proteins, generating hapten antigens that form complexes with HLA molecules in antigen-presenting cells (APCs) and are recognized by TCRs. This process activates drug-specific T cells. Antigenic drugs are covalently bound to peptides that are presented by HLA molecules to TCRs. In contrast, some drugs can bind directly and noncovalently to HLA molecules and TCRs, a binding mechanism termed the p-i concept. The TCR profile is also associated with the development of SJS/TEN. In the early stages of SJS/TEN, cytotoxic CD8^+^ T cells mainly infiltrate blister fluid and the epidermis, whereas CD4^+^ T cells predominantly infiltrate the dermis. Monocytes are present in the epidermis of TEN patients and play an important role in epidermal damage, probably by enhancing the cytotoxicity of CD8^+^ T cells. In the serum and blister fluid of SJS/TEN patients, increased levels of soluble IL-2 receptors were found and viewed as markers for activated T cells (197). These data indicate the importance of activated cytotoxic CD8^+^ T cells in the pathogenesis of SJS/TEN and the overall pathogenesis of immune-based iDILI with SJS/TEN.

## 10 Immune iDILI by ICIs

### 10.1 Basic aspects

Immunotherapy using humanized immune checkpoint inhibitors (ICIs) has emerged as a therapeutic option for patients with malignancies over the past decade (221–226). In healthy individuals, immune checkpoints are designed to moderate immune responses at a physiological level by restoring host T-cell immunity against cancer cells that have adapted toward immune evasion (221). ICIs are monoclonal antibodies that achieve immune activation by inhibiting key regulatory mechanisms, known as checkpoints, involved in cytotoxic CD8+ T-cell-mediated immunity (221–226). These monoclonal antibodies function by upregulating effector T-cell activation through inhibition of pathways that moderate their activity (221). By targeting immune checkpoint proteins, ICIs have been used in patients with metastatic melanoma, non-small cell lung cancer, pancreatic cancer, renal cell carcinoma, metastatic hormone-resistant prostate carcinoma, endometrial cancer, glioblastoma, and head and neck cancers (221, 226). The US Federal Drug Administration (FDA) approved three different categories of immune checkpoint inhibitors (ICIs) (221, 223, 226): (1) programmed cell death protein [PD-1) inhibitors (nivolumab, pembrolizumab, and cemiplimab), (2) programmed death-ligand (PDL-1) inhibitors (atezolimumab, durvalumab and avelumab), and (3) cytotoxic T-lymphocyte antigen 4 (CTLA-4) inhibitor (ipilimumab).

ICIs as monotherapy are not beneficial for all patients with a malignancy, who may then require a combination of ICIs (226). In analogy to many other treatment modalities, drug adverse reactions (ADRs) associated with ICIs are not uncommon and include immune iDILI caused by ICIs (**Table 1**) (9, 47).

### 10.2 Diagnosis

Immune iDILI by ICIs is characterized by heterogeneity among study cohorts and is found in a minority of cancer patients treated with ICIs, but a firm diagnosis is essential for good clinical management of patients experiencing this disruptive liver injury (27, 36). In this context, the use of causality assessment by robust diagnostic algorithms such as RUCAM (5–7) is strongly recommended to ascertain the diagnosis and exclude alternative causes commonly observed in cancer cohorts (27, 36). Whereas some cases were adequately evaluated for causality using RUCAM, other cases must be classified as suspected because RUCAM was not used (**Table 16**) (26, 27, 227–233).

**Table 16:** Selected immune checkpoint inhibitors implicated or not implicated in immune iDILI by ICIs as verified by RUCAM.

Immune checkpoint inhibitors	Immune iDILI by ICIs, cases (*n*)	RUCAM used as causality method	References
Adebrelimab	1	YES	Gao, 2015 (230)
Atezolizumab	1	YES	Tzadok, 2022 (26)
Atezolizumab	21	YES	Hountondji, 2024 (27)
Atezolizumab	458	NO	Liu, 2023 (229)
Atezolizumab	2	NO	Meunier, 2024 (228)
Avelumab	55	NO	Liu, 2023 (229)
Camrelizumab	12	YES	Gao, 2025 (230)
Cemiplimab	733	NO	Liu, 2023 (229)
Cemiplimab	1	NO	Meunier, 2024 (228)
Durvalumab	345	NO	Liu, 2023 (229)
Durvalumab	3	NO	Meunier, 2024 (228)
Durvalumab + other ICIs or non-ICIs	6	YES	Swanson, 2022 (232)
Ipilimumab	535	NO	Liu, 2023 (229)
Ipilimumab +Nivolumab	9	YES	Hountondji, 2023 (227)
Ipilimumab +Nivolumab	1418	NO	Liu, 2023 (229)
Nivolumab	6	YES	Hountondji, 2023 (227)
Nivolumab	29	NO	Meunier, 2024 (228)
Nivolumab + Ipilimumab	773	NO	Liu, 2023 (229)
Nivolumab + Ipilimumab	2	NO	Meunier, 2024 (228)
Nivolumab + Ipilimumab	28	YES	Hountondji, 2024 (27)
Pembrolizumab	70	YES	Tsung, 2019 (231)
Pembrolizumab	1	YES	Gao, 2025 (230)
Pembrolizumab	3	YES	Hountondji, 2023 (227)
Pembrolizumab	95	YES	Hountondji, 2024 (27),
Pembrolizumab	1169	NO	Liu, 2023 (229)
Pembrolizumab	11	NO	Meunier, 2024 (228)
Pembrolizumab + Ipilimumab	435	NO	Liu, 2023 (229)
Sintilimab	71	YES	Zheng, 2023 (233)
Sintilimab	8	YES	Gao, 2025 (230)
Tislelizumab	11	YES	Gao, 2025 (230)

Abbreviations: ICIs, immune checkpoint inhibitors; iDILI, idiosyncratic drug-induced liver injury; RUCAM, Roussel Uclaf Causality Assessment Method.

Patients with suspected immune iDILI caused by ICIs were appropriately evaluated and received the correct treatment if their cases were assessed for causality using the RUCAM (**Table 16**) (26, 27 22, 230–233). A good example of professional evaluation was a case report published under the title: *Acute Liver Failure Following a Single Dose of Atezolizumab*, with causality assessed using the updated RUCAM (26). In contrast, cases lacking RUCAM causality assessment remain problematic because the correct diagnosis may have been missed through failure to consider alternative causes (228, 229). Open questions also relate to other reports that did not consider RUCAM (234, 235). More specifically, in a best-practice paper on ICIs, RUCAM was ignored, making the so-called best practice proposals less practicable and irrelevant (234). RUCAM was also not mentioned in a safety paper, and its conclusions therefore remained vague (235), while the issue of the missing use of RUCAM was discussed in another publication (236). As a result, each patient with suspected DILI associated with the use of ICIs should be assessed using RUCAM to determine causality for the suspected drug. Indeed, patients with malignancies and increased LTs undergoing therapy with ICIs represent a challenging cohort because the diagnosis of immune iDILI may be confounded by the invasive nature of the underlying cancer and the significant comorbidities associated with advanced age, often requiring often polypharmacy. This issue was clearly outlined in a carefully performed RUCAM-based study, which showed that the most commonly identified alternative causes among 50 liver injury cases were progressive liver tumor metastasis (56%), while other etiologies included malignant biliary obstruction (4%), nonhepatic diseases (9%), and other biliary obstructions or unknown causes (221). Apart from tumor infiltration, additional alternative causes included other forms of DILI, hepatitis B and E virus infections, and missing data (227).

### 10.3 Clinical manifestations

Clinical features immune iDILI caused by ICIs are best described using data derived from studies that applied RUCAM (27, 233). Accordingly, there is a predominance of males over females by a factor of up to 1.5 (27) or 4.1 (233). Symptoms include jaundice with hepatic encephalopathy in 26.3% of cases (27).

A severity classification for immune iDILI caused by ICIs was recommended by the Common Terminology Criteria for Adverse Events (CTCAE) (237–240), but a better classification for predicting severity was suggested, aiming to include the traditional causality assessment of the updated RUCAM (27), as described in a previous report (7). Based on 100 patients presenting various iDILI patterns, with a median time to onset of 20 days after treatment with ICIs, severity gradings were inconsistent and varied significantly among the classifications used, which gave equal weight to jaundice and elevated aminotransferases (27). In this context, the efficacy of the CTCAE was verified using cases assessed by the validated updated RUCAM, which helped define characteristics of immune iDILI by ICIs that could not be identified by any other nonvalidated procedure.

### 10.4 Laboratory data

In RUCAM-based cases, serum ALT activities were up to 3,111 U/L and serum ALP activities were up to 2,459 U/L, while total bilirubin levels were up to 300 μmol/L (27). Similar results were reported in another study (233). The RUCAM-based liver injury pattern, considering serum ALT and ALP values (7), was hepatocellular in 42% of cases, cholestatic in 39%, and mixed in 19% in one study (27), whereas the corresponding proportions in another study were 23.9%, 45.1%, and 31.0%, respectively (233).

### 10.5 Liver histology

Liver histology obtained from cases with RUCAM-based assessment showed biliary injury (48.6%), interface hepatitis (13.5%), and bridging necrosis (13.5%) (27). In addition, histologic evaluation of liver specimens showed enrichment of CD8+cytotoxic T-cell acute inflammatory infiltrates and a higher proportion of mixed CD8+/CD4 T-cells (241); based on real-world experience that unfortunately did not include RUCAM-based cases (242). Biopsies of extrahepatic bile ducts revealed inflammatory infiltration of the lining epithelium (100%) (53).

### 10.6 Treatment and prognosis

Cessation of the suspected ICI is recommended as soon as the diagnosis is established (241). Corticosteroid therapy is the first-line treatment (225, 241) and commonly tailored to the severity of immune IDILI caused by ICIs (225). In steroid-refractory cases of immune iDILI caused by ICIs, mycophenolate mofetil (MMF) is commonly used as a successful second-line therapy, while third-line therapy remains controversial (225). Reintroduction of ICI immunotherapy after immune iDILI may be possible in some patients based on a case-by-case conditions and involving a multidisciplinary team. In cholestatic cases, ursodeoxycholic acid may be considered (27).

Overall prognosis depends on the severity of immune iDILI by ICIs, the efficacy achieved by immunosuppressants, and the progression of the underlying malignancy. During follow-up, 22% of patients died, mostly after progress of the cancer, and a few patients succumbed to acute liver failure (27). The 3-month lethality rate was significantly associated with iDILI and hepatic encephalopathy.

### 10.7 Molecular pathomechanisms

The pathogenesis of immune iDILI caused ICIs was broadly discussed in the literature (243–247), also considering aspects of the tumor environment, liver histology, the microbiome, and the role of hepatocytes and nonhepatocytes that become sensitized in the course of liver injury (243). However, the injurious effects can be traced back to immune activation by ICIs directed primarily against hepatocytes, which leads to T-cell mediated hepatitis and hepatocyte death (243, 244). More specifically, the activation of cytotoxic T cells that inadvertently target the liver can also modify the functions of other cells, such as B cells and T-helper cells, as well as innate immune cells including macrophages and dendritic cells, reflecting a complex interplay with cross-talk among different cell types (225). In addition, ICIs modify the tumor microenvironment and circulating chemokines and cytokine levels (243), with upregulation of interleukin (IL)-6, IL1b, interferon (IFN)-γ, tumor necrosis factor (TNF)-α, and chemokines including CXCL9, CXCL10, CXCL11, and CXCL13 (225), leading to excessive cytokine secretion in the form of a cytokine storm (225, 244). In fact, the activation of immune cells, including liver-infiltrating CD8^+^ T cells, monocytes, and macrophages, contributes to tissue inflammation, as shown in patients with immune iDILI caused by ICIs (243, 246). Finally, there may be cross-reactivity with the microbiome, hypersensitivity reactions, and a specific effect of programmed death-ligand 2 (PD-L2) (243, 247).

## 11 Diagnostic workflow for RUCAM-based refined classification of the six iDILI types

To facilitate the clinical approach, a diagnostic workflow in the form of an algorithm for the RUCAM-based refined classification of immunologic and autoimmune iDILI types is presented (**Figure 2**).

Suspected iDILI requires ALT >5× ULN and/or ALP >2× ULN, as well as the ratio (R) value for pattern classification.

At first presentation, simultaneous measurement of serum ALT and ALP activities is recommended, with each expressed as a multiple of the upper limit of the normal range (ULN).

The R value is calculated using the multiples of the ULN for ALT and ALP, providing the R-based laboratory pattern classification of iDILI.

## 12 Conclusions

This critical analysis of the current literature revealed the existence of six immune or autoimmune iDILI types, allowing for a refinement of the iDILI classification. There has been encouraging progress because cases of all types were assessed for causality using the traditional or updated RUCAM, which clearly verified the diagnosis. Based on this strong evidence, correct description of various characteristics, including clinical manifestations, genetic risk factors, laboratory results, liver histology, treatment modalities, and prognosis, were possible. Mechanistic intrahepatic steps leading to immune and autoimmune iDILI are broadly similar. In addition to non-CYP pathways, most drugs implicated in iDILI are degraded or toxified in the liver, the major metabolic organ Although most iDILI cases are likely triggered by processes involving immunologic or autoimmune pathways, exemptions must be considered. In general, immunity and autoimmunity develop through activation of the innate immune system, followed by activation of the adaptive immune system. Apart from hepatic parenchymal cells, a variety of nonparenchymal cells are involved in the evolution of iDILI. Among these different cells, there is extensive cross-talk, viewed as a complex interplay involving mediators such as interleukins.
